# Emerging Electrode Materials for Next-Generation Electrochemical Devices: A Comprehensive Review

**DOI:** 10.3390/mi17010106

**Published:** 2026-01-13

**Authors:** Thirukumaran Periyasamy, Shakila Parveen Asrafali, Jaewoong Lee

**Affiliations:** Department of Fiber System Engineering, Yeungnam University, Gyeongsan 38541, Republic of Korea

**Keywords:** electrochemical devices, energy storage, fuel cells, electrocatalysis, nanostructuring, electrode materials

## Abstract

The field of electrochemical devices, encompassing energy storage, fuel cells, electrolysis, and sensing, is fundamentally reliant on the electrode materials that govern their performance, efficiency, and sustainability. Traditional materials, while foundational, often face limitations such as restricted reaction kinetics, structural deterioration, and dependence on costly or scarce elements, driving the need for continuous innovation. Emerging electrode materials are designed to overcome these challenges by delivering enhanced reaction activity, superior mechanical robustness, accelerated ion diffusion kinetics, and improved economic feasibility. In energy storage, for example, the shift from conventional graphite in lithium-ion batteries has led to the exploration of silicon-based anodes, offering a theoretical capacity more than tenfold higher despite the challenge of massive volume expansion, which is being mitigated through nanostructuring and carbon composites. Simultaneously, the rise of sodium-ion batteries, appealing due to sodium’s abundance, necessitates materials like hard carbon for the anode, as sodium’s larger ionic radius prevents efficient intercalation into graphite. In electrocatalysis, the high cost of platinum in fuel cells is being addressed by developing Platinum-Group-Metal-free (PGM-free) catalysts like metal–nitrogen–carbon (M-N-C) materials for the oxygen reduction reaction (ORR). Similarly, for the oxygen evolution reaction (OER) in water electrolysis, cost-effective alternatives such as nickel–iron hydroxides are replacing iridium and ruthenium oxides in alkaline environments. Furthermore, advancements in materials architecture, such as MXenes—two-dimensional transition metal carbides with metallic conductivity and high volumetric capacitance—and Single-Atom Catalysts (SACs)—which maximize metal utilization—are paving the way for significantly improved supercapacitor and catalytic performance. While significant progress has been made, challenges related to fundamental understanding, long-term stability, and the scalability of lab-based synthesis methods remain paramount for widespread commercial deployment. The future trajectory involves rational design leveraging advanced characterization, computational modeling, and machine learning to achieve holistic, system-level optimization for sustainable, next-generation electrochemical devices.

## 1. Introduction

In electrochemical devices, electrodes function as essential elements that enable electron transfer throughout redox processes. These fundamental reactions underpin numerous technological applications, encompassing batteries, fuel cells, sensing systems, and electrolytic operations [1,2,3,4]. The role of electrodes extends beyond mere conductivity; they fundamentally determine the operational efficiency, functional performance, and long-term stability of electrochemical systems. The architectural design, compositional makeup, and interfacial characteristics of electrodes exert profound influence over both the kinetic pathways and thermodynamic equilibria of electrochemical transformations, consequently affecting the device’s comprehensive functionality and practical applicability.

Contemporary electrode materials encompass a diverse range of substances, including metallic elements (such as platinum, gold, and copper) [5,6], transition metal oxides (including ruthenium oxide and manganese dioxide) [7], carbonaceous materials (comprising graphite, carbon nanotubes, and graphene) [8], and electrically conductive polymers (exemplified by polyaniline and polypyrrole) [9]. Each material category presents distinctive characteristics and benefits. For example, precious metals such as platinum demonstrate exceptional catalytic performance but involve substantial economic investment, whereas carbon-based alternatives deliver satisfactory electrical conductivity while maintaining cost efficiency. Transition metal oxides frequently combine adequate conductivity with robust stability, rendering them appropriate for diverse implementations. Furthermore, conductive polymers present adjustable properties and ecological compatibility. Therefore, understanding the function and importance of various electrode materials becomes crucial for optimizing and advancing electrochemical devices throughout numerous applications, spanning energy storage systems to environmental surveillance technologies.

Traditional electrode materials, especially those employed in battery systems [10,11,12] and related electrochemical apparatus, encounter multiple obstacles that constrain their operational performance and practical utility. These materials frequently experience restricted reaction kinetics, structural and mechanical fragility, and elevated costs. During operation, electrode materials undergo substantial volumetric expansion and contraction cycles. This behavior induces mechanical strain, resulting in electrode deterioration, disruption of electrical connectivity, and eventual device malfunction [13,14]. Such phenomena may trigger parasitic reactions, architectural modifications, or alternative degradation pathways, diminishing the device’s operational lifespan and dependability.

**Scheme 1:**
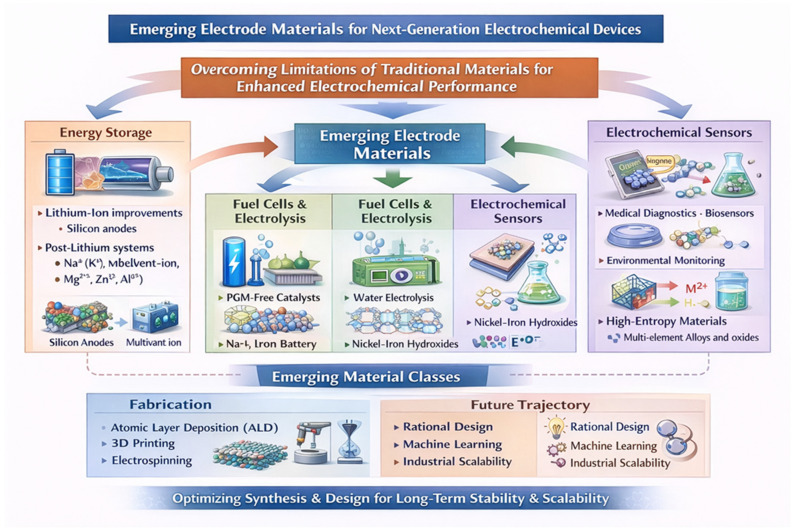
Emerging electrode materials for next-generation electrochemical devices.

Moreover, numerous conventional electrode materials depend on costly or limited elements including Pt, Pb, Ru, and similar metals, rendering them economically impractical for widespread commercial deployment [15]. Additionally, the mining and refinement of these materials frequently generate considerable environmental consequences. Nevertheless, newly emerging materials present viable solutions to these limitations by delivering innovative properties and functionalities that exceed those of traditional materials. Several distinguishing characteristics of emerging materials include enhanced reaction activity, accelerated ion diffusion kinetics, superior mechanical robustness, improved stability, environmental sustainability, and economic feasibility [15,16,17]. The advancement and implementation of novel emerging electrode materials demonstrate significant potential for progressing electrochemical technologies, facilitating the development of more efficient, dependable, and sustainable electrochemical systems. The present review distinguishes itself by providing a unified and cross-disciplinary perspective on emerging electrode materials across the full spectrum of electrochemical devices. In particular, this work systematically correlates material composition, structural architecture (from atomic to hierarchical scales), and electrochemical performance metrics across energy storage, energy conversion, and sensing applications. Furthermore, beyond summarizing recent progress, this review critically discusses stability limitations, scalability challenges, and sustainability considerations. By integrating insights from advanced materials design, fabrication strategies, and system-level optimization, this review establishes a holistic framework for guiding the rational development of next-generation electrode materials for high-performance and sustainable electrochemical devices. An extensive examination of diverse electrode materials for electrochemical devices follows in the subsequent sections. Scheme 1 shows the limitations of traditional materials and emerging electrode materials for enhanced electrochemical performance.

## 2. Electrode Materials for Energy Storage

Energy storage technologies represent critical infrastructure for modern society, addressing the intermittent nature of renewable energy sources and supporting grid stability. Electrochemical energy storage systems, particularly batteries and supercapacitors, rely fundamentally on electrode materials that govern their performance characteristics. This section provides a comprehensive overview of electrode materials organized by battery chemistry, examining both established technologies and emerging alternatives.

### 2.1. Lithium-Ion Batteries

Lithium-ion batteries have established dominance in portable electronics and electric vehicle applications due to their high energy density and operational efficiency. The performance of these systems depends critically on the electrode materials selected for both cathode and anode components.

#### 2.1.1. Currently Utilized Cathode Materials

**Figure 1:**
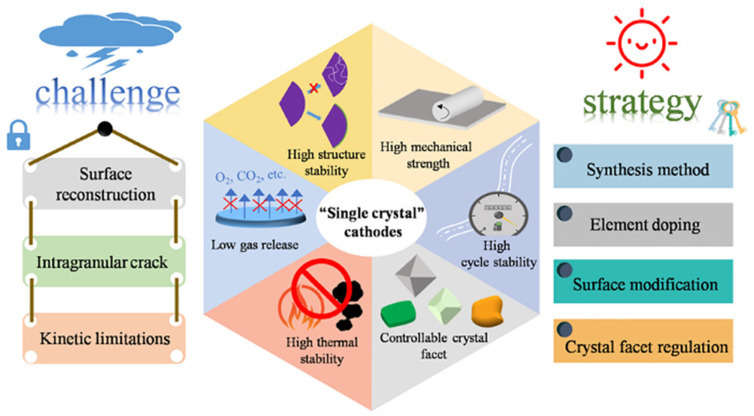
Schematic illustration of the Challenges and Strategies for optimizing “Single crystal” cathodes. Challenges include Surface reconstruction, Intragranular crack formation, and Kinetic limitations. Strategies involve Synthesis method, Element doping, Surface modification, and Crystal facet regulation. Reprinted with permission from Ref. [29]. Copyright 2021 American Chemical Society.

The cathode represents a crucial component determining the overall capacity and voltage characteristics of lithium-ion batteries (Figure 1). Traditional cathode materials include layered oxides, spinel structures, and polyanion compounds, each presenting distinct advantages and limitations. Layered oxide cathodes such as LiCoO_2_ have served as the standard cathode material for commercial lithium-ion batteries. These materials exhibit high theoretical capacity and excellent electrochemical reversibility. However, cobalt’s high cost and toxicity, combined with thermal instability at elevated states of charge, have motivated the search for alternative compositions. Spinel cathodes exemplified by LiMn_2_O_4_ provide advantages including lower cost, reduced toxicity, and three-dimensional lithium-ion diffusion pathways [18,19,20,21,22,23]. The cubic spinel structure facilitates rapid ion transport, enabling high-rate performance. Nevertheless, manganese dissolution in the electrolyte and Jahn–Teller distortion at elevated temperatures compromise cycle life and capacity retention. Polyanion cathodes such as LiFePO_4_ (lithium iron phosphate) have gained substantial commercial adoption due to their exceptional thermal stability, long cycle life, and environmental benignity. The robust phosphate framework provides structural stability throughout charge–discharge cycling. The primary limitation involves lower electronic conductivity and energy density compared to layered oxides, though carbon coating and nano-structuring strategies have substantially mitigated these concerns.

#### 2.1.2. Recent Developments in Cathode Materials

Nickel-rich layered oxides, including LiNi_0.8_Co_0.1_Mn_0.1_O_2_ (NCM811), offer enhanced capacity and reduced cobalt content compared to traditional LiCoO_2_. These materials can deliver higher energy density, making them attractive for electric vehicle applications. However, they present challenges related to structural stability and surface reactivity, particularly at high states of charge. Surface coating strategies and composition optimization continue to improve their performance and safety characteristics. Emerging cathode materials under investigation include lithium-rich layered oxides, which can deliver capacities exceeding 250 mAh/g through combined cation and anion redox mechanisms. These materials activate oxygen redox chemistry in addition to transition metal redox, enabling unprecedented capacities. However, voltage fade, hysteresis, and oxygen release during cycling present significant challenges requiring resolution before practical implementation [24,25,26,27,28,29]. Current research focuses on understanding and mitigating these degradation mechanisms through compositional modifications, surface treatments, and electrolyte engineering.

#### 2.1.3. Currently Utilized Anode Materials

Graphite anodes have served as the conventional anode material for commercial lithium-ion batteries, offering good reversibility, appropriate potential versus lithium, and reasonable cost. The layered structure of graphite accommodates lithium intercalation to form LiC_6_, corresponding to a theoretical capacity of 372 mAh/g. The well-established manufacturing processes, excellent cycle life, and stable solid electrolyte interphase (SEI) formation have made graphite the industry standard for decades. Lithium titanate (Li_4_Ti_5_O_12_) operates at approximately 1.55 V versus lithium, exhibiting essentially zero volumetric strain during cycling. This “zero-strain” characteristic enables exceptional cycle life and safety. The higher operating potential eliminates concerns regarding solid electrolyte interphase formation and lithium plating. However, the elevated potential reduces cell voltage and energy density, limiting applications to situations prioritizing safety and longevity over energy density, such as grid storage and power tools.

#### 2.1.4. Recent Developments in Anode Materials

**Figure 2:**
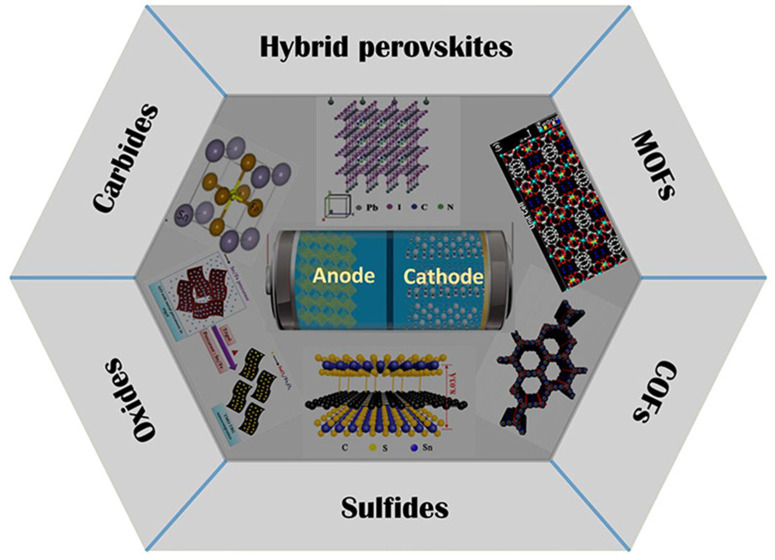
Schematic illustration of emerging electrode materials, categorized into Hybrid Perovskites, MOFs, COFs, Sulfides, Oxides, and Carbides, and their application within the anode and cathode of an electrochemical energy storage device. Reprinted with permission from Ref. [40]. Copyright 2022 American Chemical Society.

Silicon-based anodes present extraordinary theoretical capacity (approximately 4200 mAh/g for Li_22_Si_5_), representing more than tenfold improvement over graphite. This remarkable capacity has made silicon the most intensively researched next-generation anode material. However, silicon undergoes massive volume expansion (approximately 300%) during lithiation, causing mechanical pulverization, loss of electrical contact, and rapid capacity fade. Nano-structuring approaches, including silicon nanowires, nanoparticles, and porous architectures, have demonstrated improved accommodation of volume changes by providing void space and shortening lithium diffusion distances. Silicon–carbon composites, where silicon nanoparticles are embedded within conductive carbon matrices, represent a promising compromise between capacity and stability [30,31,32,33,34]. These composites buffer volume expansion while maintaining electrical connectivity. Current commercial implementations typically use 5–10% silicon content mixed with graphite, providing modest capacity improvements with acceptable cycle life. Transition metal oxides and dichalcogenides including TiO_2_, MoS_2_, and SnO_2_ have attracted attention as alternative anode materials (Figure 2). These materials can accommodate lithium through various mechanisms, including intercalation, conversion, and alloying reactions, offering capacities intermediate between graphite and silicon. TiO_2_ in various polymorphs (anatase, rutile, brookite) provides safe operation and good rate capability, though with limited capacity. MoS_2_ and other transition metal dichalcogenides offer layered structures favorable for lithium intercalation and subsequent conversion reactions, enabling higher capacities but with challenges related to volume expansion and voltage hysteresis.

#### 2.1.5. Prospects and Directions of Future Development

The future development of lithium-ion battery electrode materials focuses on several key directions, which includes the following: (i) High-Energy Cathodes: Continued development of nickel-rich and lithium-rich cathodes aims to push energy density beyond 300 Wh/kg at the cell level. This requires addressing structural stability, surface reactivity, and safety concerns through advanced coating technologies, single-crystal morphologies, and compositional gradients. (ii) High-Capacity Anodes: Silicon-dominant anodes represent the most promising path to significantly increased energy density. Future work must address cycle life limitations through advanced binder systems, prelithiation strategies, and three-dimensional architectures that accommodate volume expansion. (iii) Solid-State Compatibility: As solid-state battery technology advances, electrode materials must be adapted or redesigned for compatibility with solid electrolytes. This includes managing interfacial impedance, volume changes at rigid interfaces, and processing challenges unique to solid-state systems. (iv) Cost Reduction: Reducing or eliminating critical materials such as cobalt and developing lower-cost manufacturing processes remain essential for mass-market applications, particularly in electric vehicles and grid storage. (v) Sustainability: Increasing focus on sustainable sourcing, reduced environmental impact during manufacturing, and improved recyclability will drive materials selection and processing innovations.

### 2.2. Sodium-Ion Batteries

Sodium-ion batteries have emerged as promising alternatives to lithium-ion systems, particularly for large-scale stationary energy storage applications where cost considerations dominate. Sodium’s abundance and wide geographic distribution present significant economic advantages, though the larger ionic radius of sodium compared to lithium introduces distinct materials challenges.

#### 2.2.1. Currently Utilized Cathode Materials

Sodium-ion battery cathodes encompass several structural categories, with layered transition metal oxides representing the most extensively investigated materials. These compounds have general formula NaxMO_2_ (where M represents transition metals such as Mn, Fe, Co, or Ni) and exhibit structural similarity to lithium-ion cathodes but demonstrate distinct phase behavior during sodium extraction and insertion. The P2 and O3 structural types, distinguished by sodium coordination environment and stacking sequence, present different electrochemical characteristics. P2-type materials generally deliver superior rate capability due to larger interlayer spacing facilitating sodium diffusion, while O3-type compounds often exhibit higher volumetric energy density [35,36,37,38,39,40]. The choice between these structures involves trade-offs between power capability, energy density, and structural stability during cycling. Polyanion cathodes including sodium iron phosphate (NaFePO_4_) and sodium vanadium phosphate [Na_3_V_2_(PO_4_)_3_] offer robust structural frameworks and excellent thermal stability. The strong covalent bonds within polyanion groups stabilize the structure during cycling, enabling long operational lifetimes. However, these materials typically suffer from limited electronic conductivity, necessitating carbon coating or nanostructuring to achieve acceptable electrochemical performance. Prussian blue analogs represent a unique class of sodium-ion cathode materials characterized by open framework structures with large interstitial sites accommodating sodium ions. These materials can be synthesized through simple, aqueous-based precipitation methods at room temperature, offering significant manufacturing advantages. The general formula AxM[Fe(CN)_6_]y·nH_2_O (where A represents alkali ions and M denotes transition metals) encompasses numerous compositional variations. Despite advantages including high voltage, good rate capability, and low cost, concerns regarding water content, vacancy defects, and structural stability require ongoing attention [41,42,43].

#### 2.2.2. Recent Developments in Cathode Materials

Recent advances in sodium-ion cathode materials focus on compositional engineering to optimize performance. Multi-element substitution in layered oxides has improved structural stability and reduced undesirable phase transitions. For example, incorporating multiple transition metals (Mn, Fe, Ni, Ti) creates synergistic effects that enhance capacity retention and rate performance. In Prussian blue analogs, controlling synthesis conditions to minimize vacancy defects and water content has significantly improved electrochemical performance and cycle life. Low-defect Prussian blue compounds now demonstrate capacity retention exceeding 90% over thousands of cycles, making them commercially viable for grid storage applications [44,45,46,47,48,49]. Surface modification strategies, including protective coatings and surface doping, have addressed degradation mechanisms in various cathode materials. These approaches reduce electrolyte decomposition, suppress transition metal dissolution, and stabilize electrode-electrolyte interfaces.

#### 2.2.3. Currently Utilized Anode Materials

The larger ionic radius of sodium precludes efficient intercalation into graphite, necessitating alternative anode materials for sodium-ion batteries. Hard carbon has emerged as the leading anode material for sodium-ion batteries, delivering capacities ranging from 250 to 350 mAh/g. The disordered structure of hard carbon, featuring randomly oriented graphene layers and nanopores, accommodates sodium through multiple mechanisms including intercalation between graphene layers, adsorption on defect sites, and pore filling. The sodium storage mechanism in hard carbon remains incompletely understood, with ongoing research aimed at elucidating structure-property relationships. The precursor material, carbonization temperature, and heating rate significantly influence the resulting structure and electrochemical performance. Hard carbons derived from biomass sources offer sustainability advantages while delivering competitive performance.

#### 2.2.4. Recent Developments in Anode Materials

Transition metal oxides and sulfides such as TiO_2_, MoS_2_, and SnS_2_ function as sodium-ion anodes through conversion and alloying reactions. These materials can deliver high capacities but typically suffer from large voltage hysteresis, significant volume changes, and limited cycle life. Nanostructuring and composite formation with conductive matrices have improved performance, though challenges remain for practical implementation. Recent work on hollow and porous nanostructures has better accommodated volume expansion while maintaining electrical connectivity. Core–shell architectures with conductive outer layers and active inner cores show promise for improving both capacity and cycle stability. Phosphorus-based anodes including black phosphorus and red phosphorus exhibit high theoretical capacities (approximately 2600 mAh/g for Na_3_P) through alloying mechanisms. However, dramatic volume expansion (approximately 490% for Na_3_P formation), poor electronic conductivity, and phosphorus dissolution in electrolytes present substantial obstacles. Strategies involving phosphorus–carbon composites and protective coatings have shown promise for addressing these limitations. Organic compounds and polymers represent emerging anode materials offering advantages including flexibility, sustainability, and tunable electrochemical properties. Conjugated carbonyl compounds, carboxylates, and imides can reversibly store sodium through redox reactions involving organic functional groups. While these materials typically deliver modest capacities and moderate voltages, their environmental compatibility and structural diversity warrant continued investigation.

#### 2.2.5. Prospects and Directions of Future Development

Sodium-ion battery technology is approaching commercial viability, with several manufacturers announcing production facilities. Future development directions include the following: Performance Optimization: Achieving energy densities of 150–160 Wh/kg at the cell level would make sodium-ion batteries competitive with lithium iron phosphate systems for many applications. This requires optimizing both cathode and anode materials while improving electrolyte formulations and cell designs. Cost Reduction: Leveraging abundant materials and simplified manufacturing processes can provide 20–30% cost advantages over lithium-ion batteries. Developing supply chains for sodium-specific materials and scaling production will be essential. Low-Temperature Performance: Sodium-ion batteries naturally exhibit better low-temperature performance than lithium-ion systems due to faster ion transport kinetics. Optimizing this advantage could open applications in cold climates. Stationary Storage Focus: Grid-scale energy storage represents the most promising initial market, where weight is less critical than cost and cycle life. Sodium-ion batteries could enable more economical renewable energy integration.

### 2.3. Mono and Multi-Valent Ion Batteries

**Figure 3:**
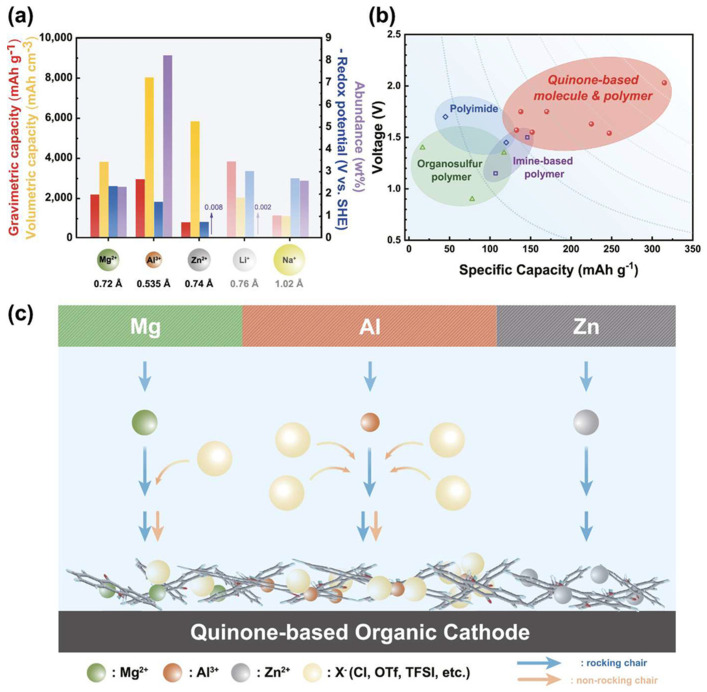
(**a**) Comparison of representative multivalent and monovalent carrier ions, noting the negative redox potentials of the metal ions versus SHE. (**b**) Operating voltage versus specific capacity for various organic cathodes. (**c**) Schematic representations of the reaction mechanisms of quinone-based organic cathodes with possible multivalent charge carriers. Reprinted with permission from Ref. [54]. Copyright 2024 American Chemical Society.

Potassium-, magnesium-, zinc-, and aluminum-ion batteries (KIBs, MIBs, ZIBs, and AIBs) have emerged as promising next-generation energy storage systems due to their cost-effectiveness, material abundance, and enhanced safety compared to lithium-ion batteries. Potassium-ion batteries benefit from potassium’s low reduction potential and small Stokes radius in organic electrolytes, which facilitate rapid ion transport and enable high-voltage operation. Magnesium- and zinc-ion systems offer dendrite-free metal anodes and high volumetric capacities (3833 and 5851 mAh cm^−3^, respectively), though the strong electrostatic interactions of multivalent ions hinder diffusion and electrode compatibility [50,51,52,53,54]. Aluminum-ion batteries, with a theoretical volumetric capacity of 8046 mAh cm^−3^, present exceptional potential for low-cost, safe storage but suffer from limited electrolyte stability and poor ion mobility (Figure 3).

#### 2.3.1. Currently Utilized Cathode Materials

For potassium-ion batteries, leading cathode candidates include Prussian blue analogs (PBAs), layered oxides (KxMO_2_), and polyanionic compounds. PBAs are particularly attractive due to their open 3D framework, which accommodates large K^+^ ions with minimal lattice strain, delivering voltages of 3.5–4.0 V and excellent cycle life [44,45,46,47,48,49]. In magnesium-ion batteries, Chevrel phases (Mo_6_S_8_) remain the most successful intercalation hosts, achieving reversible Mg^2+^ insertion with moderate capacity (80–120 mAh g^−1^) and good reversibility. Hydrated vanadium oxides (V_2_O_5_·nH_2_O) and PBAs further enhance Mg^2+^ diffusion through structural water molecules that screen electrostatic interactions. For zinc-ion systems, MnO_2_ polymorphs (α, β, δ, γ) and hydrated vanadium oxides provide capacities of 200–400 mAh g^−1^, while PBAs deliver excellent cycling stability (up to 10,000 cycles) at lower capacities (50–80 mAh g^−1^). Aluminum-ion batteries typically employ graphitic carbon cathodes, which intercalate AlCl_4_^−^ ions in ionic liquids around 2.0 V, providing long cycle life (>7500 cycles) and high-rate capability. Expanded graphite and graphene-based materials show further improvements due to enhanced ion accessibility.

#### 2.3.2. Recent Developments in Cathode Materials

Recent research focuses on defect and water control, mixed-metal substitution, and surface functionalization in PBAs to enhance structural stability and voltage profiles. Nano-structuring and composite engineering have shortened ion-diffusion paths in Mg- and Zn-based cathodes, improving rate performance and capacity retention. Additionally, organic cathodes such as conjugated carbonyl compounds and conductive polymers have gained interest for use in K- and Al-ion systems, offering mechanical flexibility and environmental sustainability despite conductivity limitations.

#### 2.3.3. Currently Utilized Anode Materials

In K-ion batteries, graphite anodes enable efficient K^+^ intercalation to form KC_8_ (279 mAh g^−1^), providing compatibility with existing lithium-ion infrastructure. Other carbon materials—soft and hard carbons—balance capacity and rate performance, though large volume expansion (~60%) during potassiation requires optimized binders and structures. For MIBs, magnesium metal anodes provide high theoretical capacity and dendrite-free deposition but demand specialized electrolytes to prevent surface passivation. Zinc metal anodes, widely used in aqueous systems, are low-cost and safe but face dendrite growth and hydrogen evolution challenges. In AIBs, aluminum metal is effectively cycled in chloroaluminate ionic liquids, allowing reversible plating/stripping without dendrites, although these electrolytes are moisture-sensitive and difficult to handle.

#### 2.3.4. Recent Developments in Anode Materials

Advancements in alloy-type anodes (Sn, Sb, P, Bi) and carbon–metal composites have mitigated mechanical stress during cycling in K- and Mg-ion batteries. Hollow nanostructures and carbon matrices improve cycle life and electrical connectivity. For zinc anodes, surface coatings, artificial solid–electrolyte interphases (SEI), and 3D current collectors (e.g., porous foams, carbon scaffolds) have effectively suppressed dendrite formation and improved reversibility. For aluminum systems, efforts are directed toward developing non-ionic liquid electrolytes and intercalation-type anodes compatible with broader operating conditions.

#### 2.3.5. Prospects and Directions of Future Development

Future progress depends on rational materials design, electrolyte optimization, and mechanistic understanding of multivalent-ion transport. K-ion batteries require refined electrolyte formulations and insight into solvation and SEI formation. MIBs need breakthroughs in wide-window, non-passivating electrolytes and fast-ion conductors. ZIBs show strong promise for grid-scale storage due to their aqueous safety and low cost, provided anode stability and cathode dissolution are addressed. AIBs demand novel electrolytes enabling reversible Al^3+^ or AlCl_4_^−^ intercalation in cost-effective, scalable systems. Continued advances in computational screening, in situ characterization, and hybrid-ion chemistries (e.g., Mg–Li or Al–air) may accelerate development toward practical multivalent-ion batteries.

#### 2.3.6. Comparative Analysis of Multivalent-Ion Batteries

Figure 3 provides a comparative overview of multivalent and monovalent carrier ions, illustrating the trade-offs between different battery chemistries. Multivalent ion batteries (magnesium, zinc, aluminum) offer theoretical advantages including higher volumetric capacity due to multiple electrons transferred per ion and enhanced safety through reduced dendrite formation. However, the strong electrostatic interactions between multivalent ions and host structures create significant kinetic barriers, presenting fundamental challenges for materials development [55,56,57,58]. The progression from monovalent (Li^+^, Na^+^, K^+^) to divalent (Mg^2+^, Zn^2+^) to trivalent (Al^3+^) ions involves increasing electrostatic interactions that severely limit ion mobility in solid hosts. This fundamental challenge requires either 1. identifying host materials with weak electrostatic interactions and large diffusion channels, 2. utilizing alternative storage mechanisms such as anion intercalation, or 3. developing hybrid systems that combine multivalent metal anodes with monovalent ion cathodes. Current research suggests that aqueous zinc-ion batteries represent the most promising near-term multivalent technology due to facile zinc deposition in aqueous electrolytes and availability of suitable cathode materials. Magnesium-ion and aluminum-ion batteries require fundamental breakthroughs in electrolyte and cathode development before practical implementation.

### 2.4. Supercapacitors

Supercapacitors (also termed electrochemical capacitors or ultracapacitors) bridge the gap between conventional capacitors and batteries, offering higher power density than batteries and greater energy density than capacitors. Energy storage in supercapacitors occurs through two primary mechanisms: electric double-layer capacitance (EDLC) and pseudocapacitance.

#### 2.4.1. Currently Utilized Materials for Electric Double-Layer Capacitors

Electric double-layer capacitors store charge electrostatically at the electrode-electrolyte interface, with capacitance proportional to surface area. Carbon materials with high surface area, good electronic conductivity, and appropriate pore structure serve as the dominant electrode materials for EDLCs. Activated carbon represents the most widely used electrode material for commercial supercapacitors, offering high surface area (1000–3000 m^2^/g), reasonable cost, and well-established manufacturing processes. The capacitance of activated carbon electrodes depends critically on surface area, pore size distribution, and electrolyte accessibility. Micropores (<2 nm) contribute significantly to surface area but may be inaccessible to electrolyte ions, while mesopores (2–50 nm) facilitate ion transport. Optimization of pore structure through activation conditions and precursor selection enables tailoring of electrochemical performance. Carbon nanotubes (CNTs) provide high electronic conductivity, mechanical strength, and accessible surface area. The one-dimensional structure of CNTs facilitates rapid electron transport and ion diffusion. Single-walled carbon nanotubes (SWCNTs) offer higher surface area than multi-walled carbon nanotubes (MWCNTs), though at greater cost. The entangled network structure of CNT electrodes provides mechanical flexibility and excellent cycle stability. However, the high cost of CNT production has limited widespread commercial adoption. Graphene represents the ultimate two-dimensional carbon material, offering theoretical surface area of 2630 m^2^/g, exceptional electronic conductivity, and mechanical robustness. However, graphene sheets tend to restack through π-π interactions, dramatically reducing accessible surface area. Strategies to prevent restacking include chemical functionalization, spacer insertion, and three-dimensional architecture formation. Reduced graphene oxide (rGO), produced through chemical reduction in graphene oxide, provides a cost-effective alternative to pristine graphene, though with somewhat reduced conductivity.

#### 2.4.2. Recent Developments in Electric Double-Layer Capacitor Materials

Carbide-derived carbons (CDCs) synthesized through selective etching of metal or metalloid atoms from carbide precursors offer exceptional control over pore size and distribution. The ability to tune pore size with sub-angstrom precision enables matching pore dimensions to electrolyte ion sizes, maximizing capacitance through optimal ion packing. CDCs have demonstrated some of the highest normalized capacitances (capacitance per unit surface area) among carbon materials [59,60,61,62,63]. Biomass-derived carbons produced from renewable precursors including coconut shells, wood, and agricultural waste offer sustainable and cost-effective alternatives to synthetic carbons. Appropriate activation and processing can yield high surface area carbons with performance comparable to commercial activated carbons. The inherent heteroatom content (nitrogen, oxygen, sulfur) in many biomass precursors can introduce pseudocapacitive contributions, enhancing overall capacitance. Three-dimensional carbon architectures including aerogels, foams, and hierarchical porous structures combine high surface area with excellent electrical conductivity and mechanical integrity. These materials provide continuous electron transport pathways and facile ion diffusion, enabling high rate capability. Additive manufacturing and templating approaches enable precise control over pore architecture and electrode geometry.

#### 2.4.3. Currently Utilized Pseudocapacitive Materials

Pseudocapacitance arises from fast, reversible faradaic reactions occurring at or near the electrode surface, providing higher specific capacitance than purely electrostatic EDLC mechanisms. Pseudocapacitive materials include transition metal oxides, conducting polymers, and certain layered materials. Transition metal oxides such as RuO_2_, MnO_2_, Co_3_O_4_, and NiO exhibit pseudocapacitance through surface or near-surface redox reactions involving protons or cations from the electrolyte. Hydrous ruthenium oxide (RuO_2_·xH_2_O) demonstrates exceptional specific capacitance (>700 F/g) and excellent conductivity, representing the benchmark pseudocapacitive material. However, ruthenium’s high cost and toxicity limit practical application. Manganese dioxide offers an economically viable alternative, with theoretical capacitance of 1370 F/g, though practical values typically range from 200 to 400 F/g due to limited conductivity and proton diffusion constraints. Nano-structuring and composite formation with conductive carbons have significantly improved manganese oxide performance. Conducting polymers including polyaniline (PANI), polypyrrole (PPy), and poly(3,4-ethylenedioxythiophene) (PEDOT) store charge through reversible doping and de-doping processes involving ion insertion and extraction. These materials offer high pseudocapacitance, good conductivity in the doped state, and facile synthesis through electrochemical or chemical polymerization [64,65,66,67]. However, conducting polymers typically suffer from limited cycle stability due to swelling, shrinking, and structural degradation during repeated doping–dedoping cycles. Composite formation with carbon materials or metal oxides has improved mechanical stability and cycle life.

#### 2.4.4. Recent Developments in Pseudocapacitive Materials

MXenes represent a recently discovered family of two-dimensional transition metal carbides and nitrides with general formula Mn + 1XnTx (where M denotes a transition metal, X represents carbon or nitrogen, and Tx indicates surface terminations). MXenes combine metallic conductivity with hydrophilic surfaces, enabling excellent electrochemical accessibility in aqueous electrolytes. The accordion-like layered structure provides facile ion intercalation, while the high volumetric capacitance (>1500 F/cm^3^) makes MXenes particularly attractive for applications requiring compact energy storage. However, oxidative degradation in aqueous environments and restacking of MXene sheets present challenges requiring ongoing research. Mixed metal oxides combining multiple transition metals offer synergistic effects that enhance conductivity, capacitance, and stability compared to single-metal oxides. For example, NiCo_2_O_4_ exhibits superior performance to either NiO or Co_3_O_4_ individually, with improved electronic conductivity and multiple redox-active sites. Hierarchical nanostructures combining pseudocapacitive materials with conductive scaffolds have addressed limitations related to conductivity and mechanical stability. Core–shell nanowires, hollow nanostructures, and three-dimensional frameworks provide high surface area while maintaining electrical connectivity and accommodating volume changes during redox reactions.

#### 2.4.5. Hybrid Supercapacitors

Hybrid supercapacitors combine EDLC and pseudocapacitive electrodes or integrate battery-type and capacitive electrodes to achieve enhanced energy density while maintaining high power capability. Common configurations include asymmetric supercapacitors pairing activated carbon with metal oxides or conducting polymers, and lithium-ion capacitors combining activated carbon cathodes with lithium-intercalating anodes. Asymmetric supercapacitors utilize different materials for positive and negative electrodes, enabling optimization of each electrode independently and expansion of the operating voltage window. For example, pairing manganese oxide (positive electrode) with activated carbon (negative electrode) in neutral aqueous electrolytes provides improved energy density compared to symmetric activated carbon devices. The challenge lies in balancing the charge storage capacities and kinetics of the two electrodes to prevent overcharging or underutilization of either electrode [68,69,70,71]. Careful electrode mass balancing and voltage window optimization are essential for maximizing performance and cycle life. Lithium-ion capacitors combine a battery-type anode (typically pre-lithiated graphite or lithium titanate) with a capacitor-type cathode (activated carbon), bridging the performance gap between supercapacitors and lithium-ion batteries. The battery-type anode provides high capacity and low potential, while the capacitor-type cathode delivers high power capability, resulting in devices with energy density approaching batteries and power density approaching supercapacitors. However, the kinetic mismatch between battery and capacitor electrodes, along with the requirement for anode pre-lithiation, complicate manufacturing and limit cycle life. Recent developments in fast-charging battery materials and optimized cell designs have improved the performance balance.

#### 2.4.6. Prospects and Directions of Future Development

Future development of supercapacitor electrode materials focuses on several key directions. Increased Energy Density: Bridging the energy density gap between supercapacitors and batteries through advanced pseudocapacitive materials, optimized hybrid configurations, and wider voltage windows will expand application opportunities. Improved Pseudocapacitive Materials: Developing pseudocapacitive materials that combine high capacitance with excellent cycle stability (>100,000 cycles) and rate capability will enable high-performance devices. Advanced Electrolytes: Ionic liquids, water-in-salt electrolytes, and redox-active electrolytes can extend voltage windows and introduce additional charge storage mechanisms, enhancing energy density. Manufacturing Scalability: Developing cost-effective, scalable synthesis and processing methods for advanced materials (graphene, MXenes, nanostructured metal oxides) will be essential for commercialization. Application-Specific Optimization: Tailoring supercapacitor designs for specific applications (automotive regenerative braking, grid frequency regulation, portable electronics) through materials selection and cell engineering will maximize performance and market adoption.

## 3. Electrode Materials for Fuel Cells

Fuel cells convert chemical energy directly into electrical energy through electrochemical reactions, offering high efficiency and low emissions. The performance of fuel cells depends critically on electrocatalyst materials that facilitate the oxygen reduction reaction (ORR) at the cathode and fuel oxidation reactions at the anode.

### 3.1. Proton Exchange Membrane Fuel Cells

**Figure 4:**
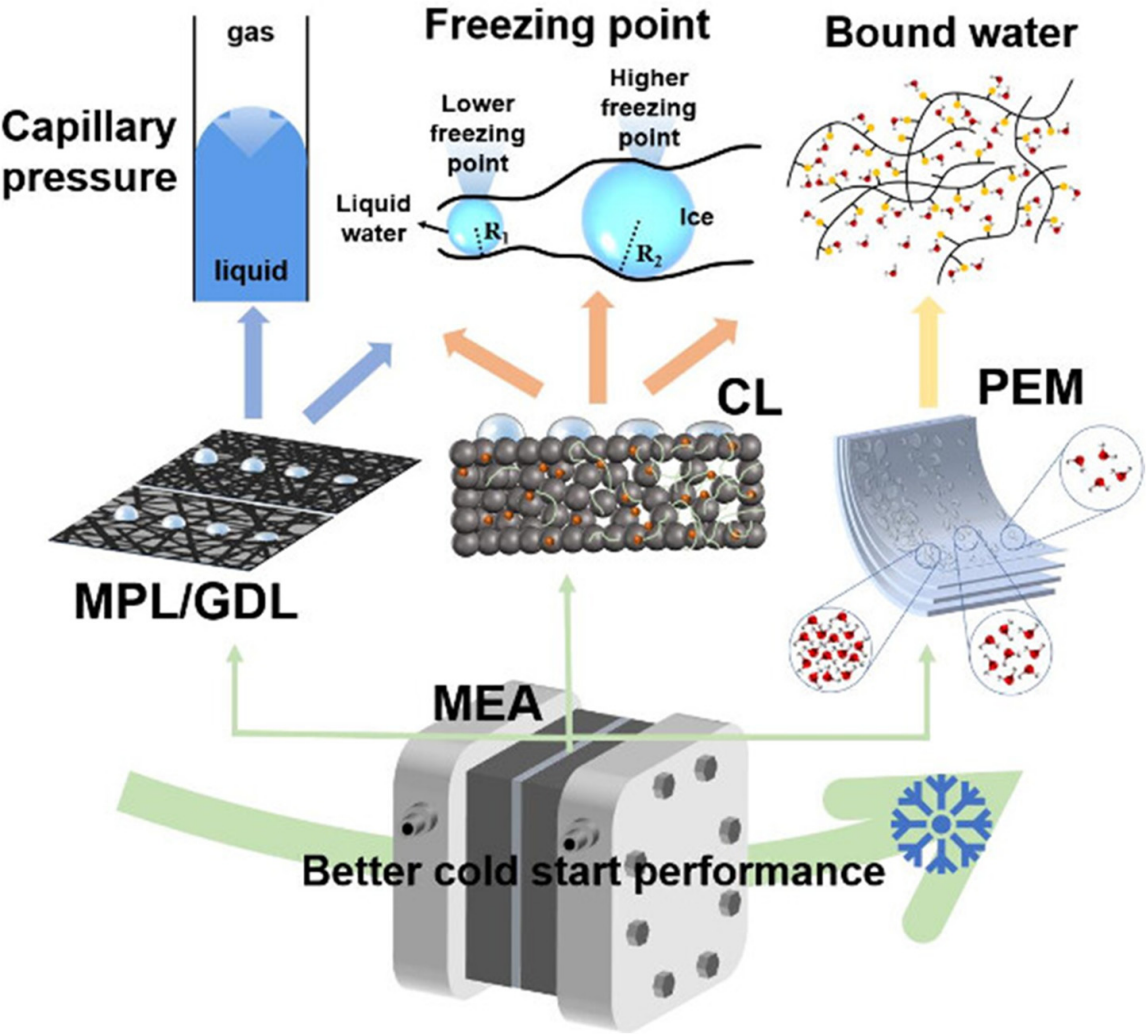
Schematic illustration of strategies for enhancing the cold-start performance of a Membrane Electrode Assembly (MEA) through the modification of its key components. Reprinted with permission from Ref. [76]. Copyright 2025 American Chemical Society.

Proton exchange membrane fuel cells (PEMFCs) operate at relatively low temperatures (60–80 °C) using a polymer electrolyte membrane. These devices are applied in transportation, portable power, and stationary power generation (Figure 4). The key electrochemical reactions involve hydrogen oxidation at the anode and oxygen reduction at the cathode. The oxygen reduction reaction at the PEMFC cathode proceeds slowly even with the best catalysts, requiring substantial catalyst loading to achieve acceptable performance. The ORR involves multiple electron transfer steps and can follow either a four-electron pathway (directly reducing O_2_ to H_2_O) or a two-electron pathway (reducing O_2_ to H_2_O_2_), with the four-electron pathway being more desirable for fuel cell applications. Platinum-based catalysts represent the state-of-the-art for ORR electrocatalysis, exhibiting the optimal balance of activity, stability, and conductivity. Platinum nanoparticles supported on high-surface-area carbon (Pt/C) constitute the standard commercial catalyst, typically with platinum loading of 0.1–0.4 mg/cm^2^. However, platinum’s high cost, limited availability, and susceptibility to poisoning and degradation motivate ongoing efforts to reduce platinum usage or develop alternative catalysts. Platinum alloys combining platinum with transition metals (such as Co, Ni, Fe, or Cu) have demonstrated enhanced ORR activity compared to pure platinum. The electronic and geometric modifications induced by alloying alter the binding strength of oxygen-containing intermediates, optimizing the reaction pathway. Pt_3_Ni and Pt_3_Co alloys have shown particularly promising performance, with mass activities (current per mass of platinum) exceeding that of pure platinum by factors of 2–4. However, the less noble alloying elements tend to dissolve preferentially during operation, leading to platinum-enriched surface layers and gradual performance degradation [72,73,74,75,76].

Core–shell structures featuring a non-platinum or platinum alloy core with a thin platinum shell offer a strategy for reducing platinum usage while maintaining high activity. The core material provides structural support and can induce electronic effects that enhance the catalytic properties of the platinum shell. For example, Pd@Pt core-shell nanoparticles with 1–2 atomic layers of platinum on palladium cores have demonstrated excellent ORR activity with substantially reduced platinum content. The challenge lies in maintaining shell integrity and preventing core dissolution during extended operation. Platinum-group-metal-free (PGM-free) catalysts represent the ultimate goal for reducing fuel cell costs and eliminating dependence on scarce platinum-group metals. The most promising PGM-free ORR catalysts include metal–nitrogen–carbon (M-N-C) materials, particularly iron–nitrogen–carbon (Fe-N-C) and cobalt–nitrogen–carbon (Co-N-C) catalysts. These materials feature atomically dispersed metal centers coordinated by nitrogen atoms within a carbon matrix, exhibiting ORR activity approaching that of platinum in some cases. However, PGM-free catalysts typically suffer from lower activity at high current densities, reduced stability (particularly in acidic environments), and susceptibility to peroxide-induced degradation. Intensive research efforts focus on understanding active site structures, improving site density, and enhancing stability through compositional and structural optimization [77,78,79].

The hydrogen oxidation reaction (HOR) at the PEMFC anode proceeds rapidly on platinum catalysts, requiring relatively low catalyst loading (typically 0.05 mg/cm^2^ or less). However, even trace amounts of impurities in the hydrogen fuel (particularly CO) can poison platinum catalysts, degrading performance. Platinum–ruthenium alloys (PtRu) demonstrate enhanced tolerance to CO poisoning compared to pure platinum, enabling operation with reformed hydrogen containing CO impurities. The bifunctional mechanism involves ruthenium facilitating oxidation of adsorbed CO through provision of oxygen-containing species at lower potentials than platinum alone. PtRu catalysts have found widespread application in direct methanol fuel cells (DMFCs) for methanol oxidation. Alternative anode catalysts including palladium-based materials and PGM-free catalysts have been explored for specific applications. In alkaline environments, nickel-based catalysts can catalyze hydrogen oxidation effectively, offering significant cost advantages. However, the requirement for polymer electrolyte membranes in PEMFCs necessitates acidic conditions, limiting the applicability of non-PGM anode catalysts.

### 3.2. Solid Oxide Fuel Cells

Solid oxide fuel cells (SOFCs) operate at elevated temperatures (600–1000 °C) using a ceramic oxide-ion-conducting electrolyte. The high operating temperature enables use of non-precious metal catalysts and allows internal reforming of hydrocarbon fuels. SOFCs find applications in stationary power generation and combined heat and power systems. SOFC cathodes must catalyze the oxygen reduction reaction while conducting both electrons and oxide ions. The triple-phase boundary (where gas phase, electronic conductor, and ionic conductor meet) represents the active site for oxygen reduction. Lanthanum strontium manganite (LSM, La_1−x_Sr_x_MnO_3_) has served as the conventional SOFC cathode material for high-temperature operation (800–1000 °C). LSM exhibits excellent electronic conductivity and structural stability but limited oxide ion conductivity, restricting oxygen reduction to triple-phase boundaries. The performance of LSM cathodes depends critically on microstructure and interfacial contact with the electrolyte. Mixed ionic-electronic conductors (MIECs) including lanthanum strontium cobalt ferrite (LSCF, La_1−x_Sr_x_Co_1−y_Fe_y_O_3−δ_) and barium strontium cobalt ferrite (BSCF, Ba_1−x_Sr_x_Co_1−y_Fe_y_O_3−δ_) conduct both electrons and oxide ions, enabling oxygen reduction across the entire electrode surface rather than only at triple-phase boundaries. This dramatically increases the active area and improves performance, particularly at reduced operating temperatures (600–800 °C). However, MIEC cathodes may react with common electrolyte materials (such as yttria-stabilized zirconia) at high temperatures, necessitating the use of interlayers or alternative electrolyte materials. Ruddlesden–Popper phases such as Ln_2_NiO_4+δ_ (where Ln represents lanthanides) exhibit high oxygen diffusivity through interstitial oxygen sites, providing excellent ORR activity at intermediate temperatures. These materials demonstrate particular promise for reducing SOFC operating temperatures while maintaining acceptable performance. SOFC anodes must catalyze fuel oxidation reactions while providing pathways for electron conduction, ion conduction, and gas diffusion. The conventional nickel–yttria-stabilized zirconia (Ni-YSZ) cermet anode has proven highly effective for hydrogen fuel but faces challenges with hydrocarbon fuels [80,81,82,83,84].

Nickel-based cermets combining nickel metal with oxide-ion-conducting ceramics (typically YSZ or gadolinia-doped ceria) provide the standard SOFC anode structure. Nickel supplies electronic conductivity and catalytic activity, while the ceramic phase conducts oxide ions and provides structural support. The interpenetrating network structure creates extensive triple-phase boundaries for fuel oxidation. However, nickel-based anodes suffer from several limitations, including carbon deposition when operating on hydrocarbon fuels, sulfur poisoning, and redox instability (dimensional changes during oxidation–reduction cycles). Alternative anode materials including mixed ionic–electronic conductors, particularly doped cerias and titanates, offer improved tolerance to carbon deposition and sulfur poisoning. Strontium titanate-based anodes (such as La-doped SrTiO_3_) demonstrate excellent redox stability and coking resistance but typically exhibit lower conductivity and catalytic activity than nickel-based materials [85,86,87]. Composite anodes combining MIEC oxides with small amounts of nickel or noble metal catalysts represent a promising compromise between activity and stability.

### 3.3. Direct Alcohol Fuel Cells

Direct alcohol fuel cells (DAFCs), particularly direct methanol fuel cells (DMFCs) and direct ethanol fuel cells (DEFCs), oxidize liquid alcohols directly at the anode, eliminating the need for fuel reforming. The liquid fuel storage provides advantages for portable applications, though alcohol crossover through the membrane and sluggish oxidation kinetics present challenges. Methanol and ethanol oxidation reactions involve multiple electron transfers and C-H bond breaking, requiring effective electrocatalysts to achieve acceptable reaction rates. Platinum–ruthenium catalysts represent the state-of-the-art for methanol oxidation, with the bifunctional mechanism and electronic effects of ruthenium enhancing activity and CO tolerance. The optimal Pt:Ru ratio typically falls around 1:1 to 2:1, balancing methanol dehydrogenation (favored by platinum) and CO oxidation (facilitated by ruthenium). Platinum-tin catalysts have shown promise for ethanol oxidation, with tin promoting C-C bond breaking required for complete ethanol oxidation to CO_2_. However, ethanol oxidation remains more challenging than methanol oxidation due to the difficulty of cleaving the C-C bond, resulting in incomplete oxidation and reduced efficiency. Palladium-based catalysts have attracted attention for alcohol oxidation in alkaline media, where palladium exhibits activity comparable to platinum at significantly lower cost. Palladium alloys with metals such as Au, Ag, Ni, and Co have demonstrated enhanced activity and stability for methanol and ethanol oxidation in alkaline environments [88,89,90,91].

## 4. Electrode Materials for Electrolysis

**Figure 5:**
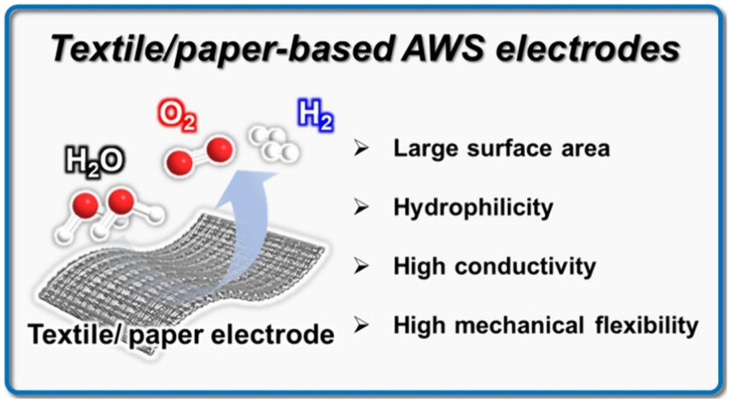
Emerging Electrocatalytic Textile Electrodes for Highly Efficient Alkaline Water Electrolysis: A Schematic View Reprinted with permission from Ref. [92]. Copyright 2024 American Chemical Society.

Electrolysis processes convert electrical energy into chemical energy, enabling production of hydrogen, oxygen, and various chemicals. Efficient electrocatalysts for both the oxygen evolution reaction (OER) and hydrogen evolution reaction (HER) are essential for viable electrolysis technologies. Water electrolysis splits water into hydrogen and oxygen, providing a pathway for storing renewable electrical energy as chemical fuel (Figure 5). The overall reaction involves the HER at the cathode and the OER at the anode, with the OER typically representing the rate-limiting step due to its sluggish kinetics involving four electron transfers [92].

### 4.1. Oxygen Evolution Reaction Catalysts

**Figure 6:**
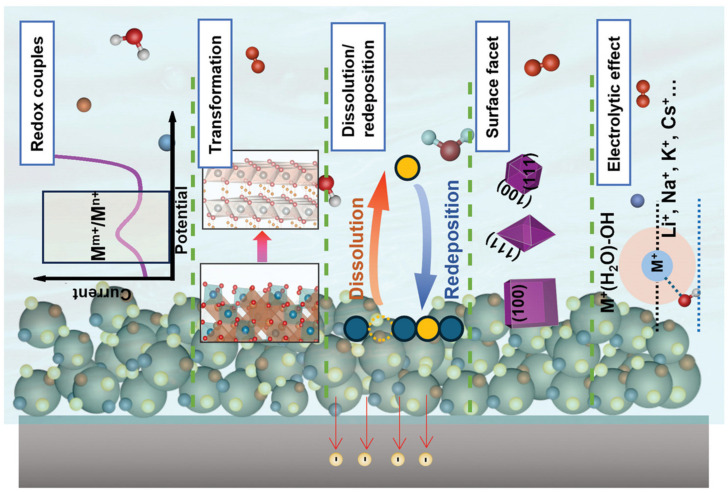
Schematic representation of the elementary process at the electrode/electrolyte interface during the Oxygen Evolution Reaction (OER). This diagram illustrates the key mechanistic steps and interfacial phenomena Reprinted with permission from Ref. [93]. Copyright 2025 Small.

The OER proceeds through multiple intermediate steps involving adsorbed hydroxide, oxygen, and hydroperoxide species, as illustrated in Figure 6. Efficient OER catalysts must bind these intermediates with optimal strength—not too strong (which would block active sites) nor too weak (which would result in slow reaction rates). Iridium and ruthenium oxides represent the most active OER catalysts for acidic conditions (required in proton exchange membrane electrolyzers). Iridium oxide demonstrates better stability than ruthenium oxide, which tends to dissolve and degrade during operation. However, the high cost and scarcity of iridium motivate efforts to reduce loading or develop alternative catalysts. Strategies include nano-structuring to maximize surface area, alloying with more abundant metals, and supporting conductive substrates. Nickel–iron hydroxides and oxyhydroxides exhibit excellent OER activity in alkaline environments, representing the benchmark catalysts for alkaline water electrolysis. The synergistic interaction between nickel and iron enhances activity beyond that of either element alone, with iron incorporation dramatically improving performance even at low concentrations. The optimal Ni:Fe ratio typically falls around 3:1 to 4:1. These materials offer significant cost advantages over noble metal catalysts, though they are restricted to alkaline conditions [90,91,92,93].

Perovskite oxides with general formula ABO_3_ (where A represents rare earth or alkaline earth elements and B denotes transition metals) provide tunable OER activity through compositional variations. Barium strontium cobalt ferrite (BSCF) and related compositions have demonstrated activity approaching that of iridium oxide in alkaline media. The ability to adjust both A-site and B-site compositions enables optimization of electronic structure, oxygen vacancy concentration, and surface properties. Transition metal phosphides, sulfides, and selenides have emerged as promising OER catalysts, particularly for alkaline electrolysis. Nickel phosphides, cobalt phosphides, and iron phosphides exhibit good activity and stability. The mechanisms by which these materials catalyze the OER remain under investigation, with evidence suggesting surface oxidation to form active oxyhydroxide species under operating conditions.

### 4.2. Hydrogen Evolution Reaction Catalysts

**Figure 7:**
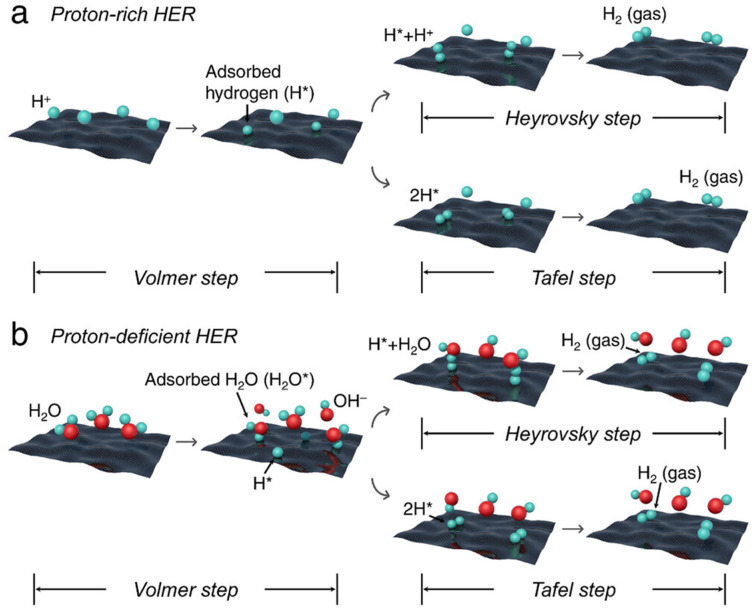
Mechanistic schemes detailing the Hydrogen Evolution Reaction (HER) pathways on electrocatalyst surfaces, comparing the reaction kinetics in (**a**) acidic (proton-rich) and (**b**) alkaline (proton-deficient) media. Reprinted with permission from Ref. [94]. Copyright 2025 Small.

The HER involves either the Volmer–Heyrovsky mechanism (two steps) or the Volmer–Tafel mechanism (two steps), as depicted in Figure 7, with the optimal pathway depending on catalyst properties and pH. Efficient HER catalysts must facilitate proton adsorption and hydrogen desorption with minimal overpotential. Platinum represents the benchmark HER catalyst, exhibiting near-optimal hydrogen binding energy and exceptional exchange current density. However, platinum’s cost motivates development of alternative materials, particularly for large-scale hydrogen production applications. Transition metal phosphides including nickel phosphide (Ni_2_P), cobalt phosphide (CoP), and molybdenum phosphide (MoP) have demonstrated excellent HER activity in both acidic and alkaline environments. These materials exhibit metallic conductivity and hydrogen binding energies approaching that of platinum. The phosphorus component modifies the electronic structure of the metal, creating active sites with favorable hydrogen adsorption properties. Transition metal dichalcogenides particularly molybdenum disulfide (MoS_2_) and tungsten disulfide (WS_2_), have attracted extensive attention as HER catalysts. The edge sites of MoS_2_ exhibit high HER activity, while the basal planes are relatively inert. Strategies to enhance activity include increasing edge site density through nano-structuring, creating sulfur vacancies on basal planes, and phase engineering (converting from semiconducting 2H phase to metallic 1T phase).

Transition metal carbides and nitrides such as molybdenum carbide (Mo_2_C) and tungsten carbide (WC) exhibit platinum-like electronic structure and demonstrate good HER activity. These materials offer improved stability compared to some phosphides and sulfides, particularly in acidic environments. However, surface oxidation during operation can reduce activity, necessitating careful control of operating conditions. Nickel–molybdenum alloys have shown remarkable HER activity in alkaline media, with activity approaching that of platinum. The synergistic effect between nickel and molybdenum creates active sites with optimal hydrogen binding energy. These materials offer significant cost advantages and demonstrate excellent stability in alkaline electrolytes [89,90,91,92,93,94].

### 4.3. CO_2_ Reduction Catalysts

**Figure 8:**
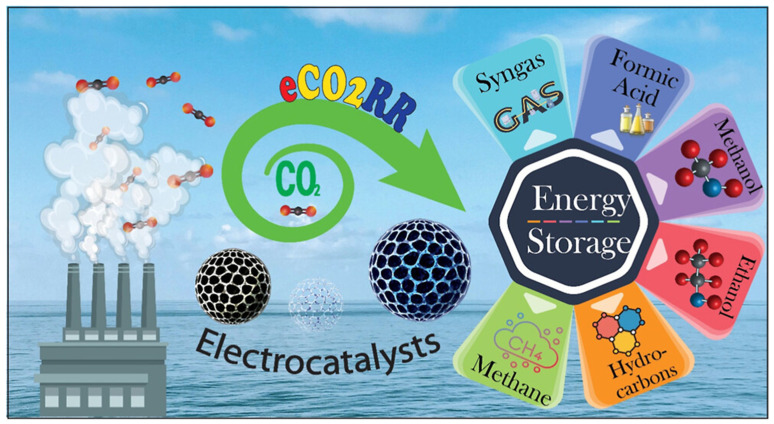
Electrochemical CO_2_ Reduction: A Scheme for Sustainable Energy Conversion and Storage. Reprinted with permission from Ref. [100]. Copyright 2025 American Chemical Society.

**Figure 9:**
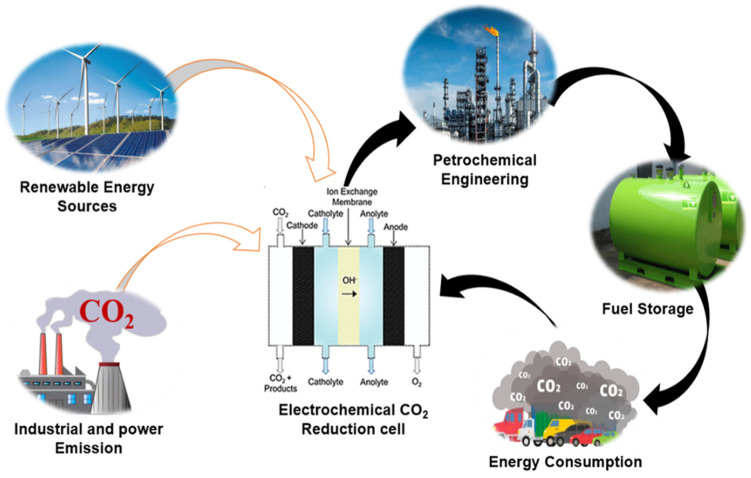
Schematic illustrating the conversion of carbon dioxide CO_2_ into valuable chemical products and fuels via an electrochemical process driven by renewable power. Reprinted with permission from Ref. [100]. Copyright 2025 American Chemical Society.

Electrochemical CO_2_ reduction converts carbon dioxide into valuable chemicals and fuels, offering a pathway for carbon utilization and storage of renewable electricity (Figure 8 and Figure 9). However, CO_2_ reduction involves multiple possible products (CO, formate, methane, ethylene, ethanol, and others), requiring selective catalysts to achieve high selectivity for desired products. Reduction in CO_2_ to CO involves a two-electron transfer and represents one of the more selective CO_2_ reduction reactions. CO serves as a valuable chemical feedstock and can be combined with H_2_ to produce synthetic fuels through Fischer–Tropsch synthesis. Gold and silver catalysts exhibit high selectivity for CO_2_ reduction to CO with minimal hydrogen evolution. Gold nanoparticles demonstrate particularly high selectivity (>90%) and activity, with performance depending on particle size and morphology. Silver offers similar selectivity at lower cost, though with somewhat reduced activity. The mechanisms involve CO_2_ adsorption and activation, followed by proton-coupled electron transfer to form adsorbed CO, which desorbs readily from gold and silver surfaces. Molecular catalysts including metal porphyrins, metal phthalocyanines, and other coordination complexes can catalyze selective CO_2_ reduction to CO. Iron and cobalt porphyrins have demonstrated particularly promising performance [95,96,97,98,99,100]. These molecular catalysts can be immobilized on electrode surfaces or incorporated into polymeric frameworks to create heterogeneous electrocatalysts combining the selectivity of molecular catalysts with the stability and conductivity of solid electrodes. Metal–organic frameworks (MOFs) and covalent organic frameworks (COFs) featuring catalytically active metal centers provide porous structures with high densities of active sites. The tunable pore structures can enhance CO_2_ adsorption and concentration, while the periodic arrangement of active sites enables high catalytic activity. However, the limited electrical conductivity of many MOFs and COFs necessitates strategies such as incorporation of conductive additives or growth on conductive substrates.

### 4.4. Catalysts for CO_2_ Reduction to Hydrocarbons and Alcohols

Reduction in CO_2_ to multi-carbon products such as ethylene and ethanol represents a more challenging but potentially more valuable transformation, requiring C-C coupling in addition to multiple electron and proton transfers. Copper catalysts uniquely demonstrate significant activity for CO_2_ reduction to hydrocarbons and alcohols, with product distributions depending on catalyst morphology, oxidation state, and electrolyte composition. Copper’s intermediate CO binding strength (stronger than gold or silver but weaker than platinum) enables both CO_2_ activation and subsequent C-C coupling. Oxide-derived copper catalysts, produced by reducing copper oxide precursors, have shown enhanced selectivity for multi-carbon products compared to metallic copper, attributed to grain boundaries, defects, and residual subsurface oxygen. Copper alloys and composites combining copper with other metals can tune product selectivity. For example, Cu-Ag and Cu-Au catalysts have demonstrated enhanced ethylene selectivity, while Cu-Sn and Cu-In catalysts favor formate production. The secondary metal modifies the local electronic structure and CO coverage, influencing the C-C coupling probability and subsequent reduction pathways. Single-atom catalysts featuring isolated metal atoms (such as Ni, Fe, or Co) anchored on nitrogen-doped carbon supports have shown promise for selective CO_2_ reduction. While these catalysts typically produce CO or formate rather than multi-carbon products, they offer high selectivity and efficient metal utilization [101,102,103,104,105,106]. The well-defined coordination environment of single-atom sites enables fundamental studies of reaction mechanisms and structure–activity relationships.

## 5. Electrode Materials for Sensors

Electrochemical sensors detect and quantify analytes through electrochemical reactions at electrode surfaces, finding applications in medical diagnostics, environmental monitoring, and industrial process control. The electrode material determines sensor sensitivity, selectivity, response time and stability.

### 5.1. Enzyme-Based Biosensors

Enzyme-based biosensors utilize enzymes as recognition elements, exploiting their high specificity for target analytes. The enzyme catalyzes a reaction involving the analyte, producing an electrochemically detectable product or consuming an electroactive species. Glucose sensors represent the most commercially successful biosensors, with applications in diabetes management. Glucose oxidase (GO_x_) catalyzes glucose oxidation, producing gluconic acid and hydrogen peroxide. First-generation glucose sensors detect hydrogen peroxide at a platinum electrode, while second-generation sensors employ redox mediators (such as ferrocene derivatives) to shuttle electrons from GO_x_ to the electrode. Third-generation sensors achieve direct electron transfer between GO_x_ and the electrode through appropriate enzyme orientation and electrode modification.

**Figure 10:**
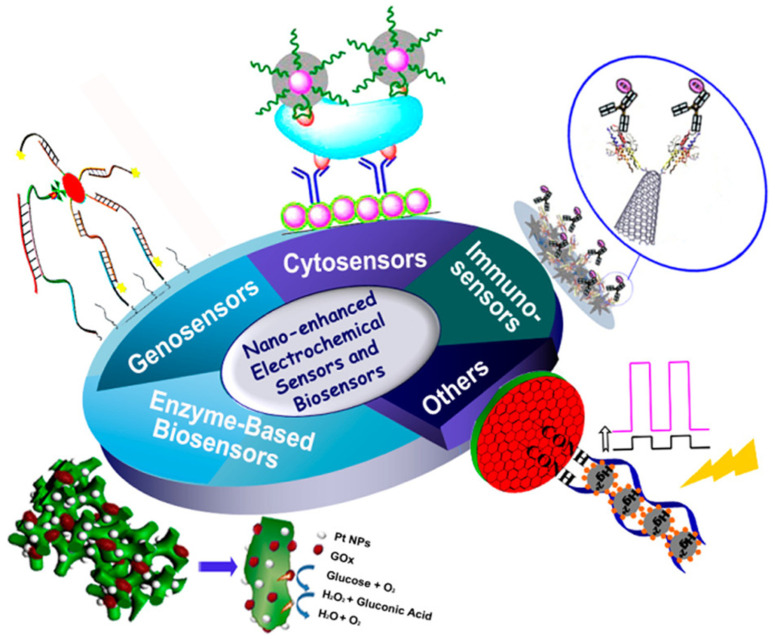
Schematic showing nanomaterial- and nanostructure-based electrochemical sensors for small molecules, biosensors (enzyme, gene, immune), and cytosensors. Reprinted with permission from Ref. [112]. Copyright 2014 American Chemical Society.

Electrode materials for enzyme immobilization must provide stable enzyme attachment while maintaining enzyme activity and facilitating electron transfer (Figure 10). Carbon-based materials including carbon nanotubes, graphene, and carbon black offer high surface area, good conductivity, and compatibility with enzyme immobilization. Metal nanoparticles (particularly gold and platinum) can enhance electron transfer and provide attachment points for enzyme immobilization through thiol linkages. Conducting polymers enable enzyme entrapment during electro-polymerization, creating integrated enzyme–polymer films [107,108,109,110,111,112].

### 5.2. Non-Enzymatic Sensors

Non-enzymatic sensors detect analytes through direct electrochemical reactions at electrode surfaces, eliminating issues associated with enzyme stability and offering advantages for harsh environments. Noble metal electrodes including platinum, gold, and palladium can catalyze direct oxidation or reduction in various analytes. For example, platinum electrodes enable non-enzymatic glucose detection through direct glucose oxidation, though with reduced selectivity compared to enzyme-based sensors. Surface modification through nano-structuring or alloying can enhance activity and selectivity.

Metal oxide and hydroxide electrodes, particularly nickel hydroxide, copper oxide, and cobalt oxide, demonstrate electrocatalytic activity for oxidation of various organic molecules including glucose, alcohols, and amino acids in alkaline media. These materials offer good stability and can be fabricated through simple electrodeposition or chemical synthesis methods. Carbon-based electrodes modified with heteroatoms (nitrogen, boron, sulfur) or functional groups exhibit enhanced electrocatalytic activity for various analytes. For example, nitrogen-doped carbon materials demonstrate improved activity for oxygen reduction and can serve as sensing platforms for hydrogen peroxide, glucose, and other species [113,114,115].

### 5.3. Heavy Metal Ion Sensors

**Figure 11:**
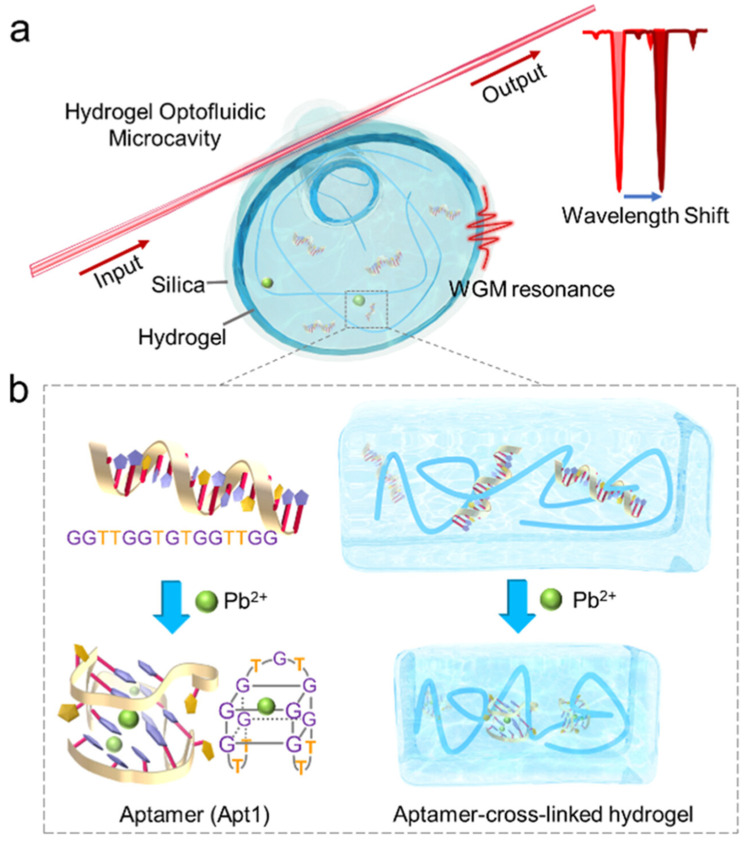
Schematic of the aptamer-cross-linked hydrogel optofluidic microcavity sensor. (**a**) The aptamer-functionalized hydrogel on the microcavity’s inner surface detects heavy metal ions, using the WGM spectrum for transduction and a fiber taper for optical signal detection. (**b**) Exposure to heavy metal ions (e.g., Pb) triggers a conformational change in the aptamer from a random coil to a G-quadruplex structure, leading to hydrogel shrinkage and a measurable red shift in resonance wavelength. Reprinted with permission from Ref. [118]. Copyright 2025 American Chemical Society.

Detection of heavy metal ions (such as Pb^2+^, Cd^2+^, Hg^2+^ and As^3+^) in water represents an important environmental and health application (Figure 11). Anodic stripping voltammetry (ASV) provides a sensitive technique for heavy metal detection, involving electrochemical preconcentration followed by anodic stripping. Bismuth film electrodes have emerged as environmentally friendly alternatives to mercury electrodes for heavy metal detection. Bismuth forms alloys with many heavy metals, enabling effective preconcentration, while the well-defined stripping peaks provide sensitive and selective detection. Bismuth films can be deposited on various substrates including glassy carbon, screen-printed carbon, and carbon paste electrodes. Carbon-based electrodes modified with metal nanoparticles, metal oxides, or functional groups demonstrate enhanced sensitivity for heavy metal detection. The high surface area and adsorptive properties of carbon materials facilitate preconcentration, while surface modifications can improve selectivity for specific metal ions [116,117,118].

## 6. Emerging Electrode Materials and Fabrication Strategies

The rapid evolution of electrochemical devices demands innovative electrode materials that transcend the performance limitations of conventional systems. While traditional materials such as graphite anodes, lithium cobalt oxide cathodes, activated carbons, and platinum-based catalysts have enabled widespread commercialization, emerging materials offer transformative opportunities for enhanced energy density, power capability, durability, sustainability, and cost-effectiveness. This section comprehensively reviews cutting-edge electrode materials and fabrication strategies across the entire spectrum of electrochemical devices, including advanced batteries, supercapacitors, fuel cells, electrolyzers, electrochemical synthesis systems, sensors, and flow batteries. The emphasis is placed on materials developed or significantly advanced within the past three years (2023–2025), representing the true frontier of next-generation electrochemical technology.

### 6.1. Advanced Two-Dimensional Materials Beyond Graphene

Two-dimensional (2D) materials have emerged as a transformative class of electrode materials, offering exceptional surface-to-volume ratios, tunable electronic properties, and unique electrochemical characteristics that surpass conventional bulk materials. While graphene established the foundation for 2D material research, recent advances in transition metal dichalcogenides (TMDs), MXenes, phosphorene, borophene, and other 2D materials have opened new frontiers for electrochemical applications.

#### 6.1.1. Mxenes

MXenes, a family of two-dimensional transition metal carbides, nitrides, and carbonitrides with the general formula Mn + 1XnTx (where M = transition metal, X = C or N, and Tx = surface terminations such as -O, -OH, -F), have rapidly emerged as one of the most promising electrode material classes for next-generation electrochemical devices [119,120,121]. The most extensively studied MXene, Ti_3_C_2_Tx, exhibits metallic conductivity (up to 24,000 S cm^−1^), hydrophilic surfaces facilitating rapid ion transport, and tunable surface terminations enabling property optimization for specific applications.

In energy storage applications, MXenes demonstrate exceptional performance in batteries and supercapacitors due to their high volumetric capacitance (1500 F cm^−3^ for Ti_3_C_2_Tx), rapid ion intercalation kinetics, and mechanical flexibility. For lithium-ion batteries, MXene-based anodes offer theoretical capacities exceeding 400 mAh g^−1^ with superior rate capability compared to graphite. In sodium-ion batteries, MXenes provide fast Na^+^ diffusion pathways and stable cycling performance, addressing the critical challenge of sluggish ion transport in Na-based systems [119]. MXene@metal–organic framework (MOF) hybrids have demonstrated synergistic effects, combining the high conductivity of MXenes with the high surface area and tailored porosity of MOFs, resulting in supercapacitor electrodes with enhanced energy density while maintaining excellent power characteristics [122,123]. In electrocatalysis, MXenes serve as exceptional supports for single-atom catalysts (SACs) and as active electrocatalysts themselves for hydrogen evolution reaction (HER), oxygen reduction reaction (ORR), and CO_2_ reduction [120,121,124]. The tunable surface terminations of MXenes enable optimization of binding energies for reaction intermediates, while their metallic conductivity ensures efficient electron transfer. MXene-derived heterostructures with transition metal oxides or chalcogenides exhibit superior electrocatalytic activity compared to pristine materials, with MXene/NiFe-layered double hydroxide composites achieving overpotentials as low as 220 mV at 10 mA cm^−2^ for oxygen evolution reaction (OER) [121,123].

Despite remarkable progress, several challenges limit widespread MXene adoption. Oxidative instability in ambient conditions requires protective strategies such as surface modification or composite formation. Restacking of MXene sheets reduces accessible surface area and ion transport kinetics, necessitating spacer insertion or 3D architecture design. Scalable synthesis beyond laboratory-scale production remains a critical bottleneck, with current etching processes involving hazardous hydrofluoric acid. Future research directions include development of fluorine-free synthesis routes, atomic-level control of surface terminations for targeted applications, integration with other 2D materials in heterostructures, and comprehensive lifecycle assessment for sustainable production [119,120,121].

#### 6.1.2. Transition Metal Dichalcogenides (TMDs)

TMDs with the general formula MX_2_ (M = Mo, W, Ti, Nb, etc.; X = S, Se, Te) exhibit layered structures with van der Waals gaps between layers, enabling facile ion intercalation and providing abundant edge sites for catalysis. The electronic properties of TMDs span from semiconducting (2H-MoS_2_, WS_2_) to metallic (1T-MoS_2_, VS_2_), offering versatility for diverse electrochemical applications. The polymorphic nature of TMDs enables performance tuning through phase engineering. The semiconducting 2H phase of MoS_2_ can be converted to the metallic 1T phase through chemical or electrochemical methods, dramatically enhancing electrical conductivity and creating additional active sites for electrocatalysis [121]. Metastable 1T-MoS_2_ exhibits HER activity approaching that of platinum in acidic media, with Tafel slopes as low as 40 mV dec^−1^ and exchange current densities exceeding 10^−4^ A cm^−2^.

In case of energy storage applications, TMDs serve as high-capacity electrode materials for alkali-ion batteries through conversion and intercalation mechanisms. MoS_2_ anodes deliver theoretical capacities of 670 mAh g^−1^ for lithium-ion batteries and demonstrate excellent rate performance when nanostructured or hybridized with conductive matrices. Ternary metal sulfides combining multiple transition metals (e.g., NiCo_2_S_4_, ZnCo_2_S_4_) offer enhanced electronic conductivity and multiple redox reactions, achieving specific capacities exceeding 1000 mAh g^−1^ for sodium-ion battery anodes [125]. In electrocatalysis, the catalytic activity of TMDs originates primarily from edge sites and sulfur vacancies, which can be enhanced through defect engineering, heteroatom doping, and nanostructuring. WS_2_ nanosheets with engineered sulfur vacancies demonstrate bifunctional activity for both HER and OER, enabling application in overall water splitting. TMD/graphene heterostructures leverage the high conductivity of graphene and the catalytic activity of TMD edges, achieving synergistic performance enhancement [121].

#### 6.1.3. Phosphorene, Borophene, and Beyond

Few-layer black phosphorus (phosphorene) exhibits a puckered honeycomb structure with thickness-dependent bandgap and anisotropic properties. Phosphorene demonstrates theoretical lithium storage capacity of 2596 mAh g^−1^ and exceptional rate capability due to low ion diffusion barriers. However, ambient instability and synthetic challenges have limited practical applications, driving research toward surface passivation strategies and phosphorene-based composites [119]. Two-dimensional boron sheets (borophene) possess metallic conductivity, mechanical flexibility, and high theoretical capacity for metal-ion storage. Borophene exhibits promising HER activity with near-optimal hydrogen binding energy. Synthesis challenges, including substrate requirements and ambient instability, remain significant obstacles to widespread investigation and application. Layered double hydroxides (LDHs), with the general formula [M^2+^_1−*x*_M^3+^*_x_*(OH)_2_]*^x^*^+^(A^n−^)_*x*/n_·mH_2_O consist of positively charged brucite-like layers with intercalated anions. The compositional flexibility enables incorporation of various divalent (Mg, Ni, Co, Zn) and trivalent (Al, Fe, Cr) metals, allowing property tuning for specific applications. NiFe-LDH has emerged as one of the most active non-precious OER catalysts, achieving performance comparable to or exceeding IrO_2_ benchmarks. LDHs serve as excellent precursors for mixed metal oxides, hydroxides, and sulfides through calcination or electrochemical activation, providing hierarchical structures with enhanced surface area and conductivity [123].

### 6.2. Single-Atom Catalysts

**Figure 12:**
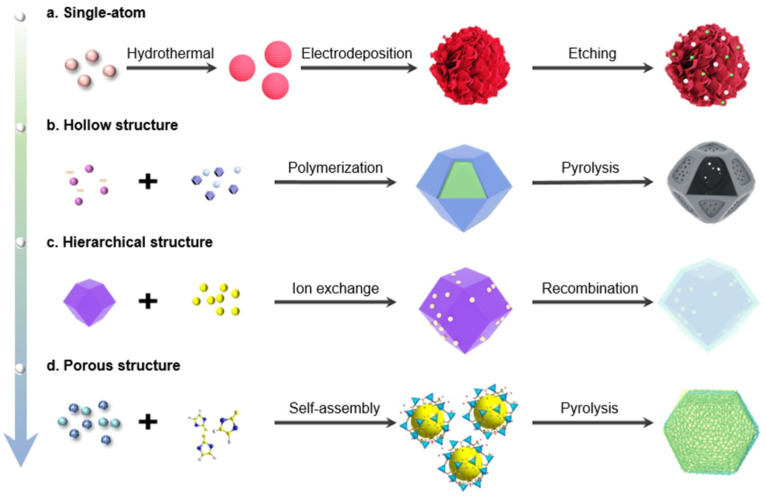
Common synthesis methods for SACs featuring various framework architectures and structures Reprinted with permission from Ref. [130]. Copyright 2025 Elsevier.

Single-atom catalysts (SACs) represent the ultimate limit of catalyst downsizing, featuring isolated metal atoms dispersed on support materials at sub-nanometer scale (Figure 12). SACs maximize atom utilization efficiency, provide well-defined active sites for mechanistic studies, and exhibit unique electronic properties distinct from nanoparticles or bulk materials. The field of SACs has experienced explosive growth in recent years, with applications expanding across all major electrochemical reactions [126,127,128,129,130].

#### 6.2.1. Metal-Nitrogen-Carbon (M-N-C) Catalysts

M-N-C catalysts featuring atomically dispersed transition metal centers (Fe, Co, Ni, Mn, Cu) coordinated by nitrogen atoms in carbon matrices have emerged as the most promising platinum-group-metal-free (PGM-free) catalysts for ORR in fuel cells and metal-air batteries [126]. The well-defined M-N_x_ coordination environment (typically M-N_4_ or M-N_2+2_ configurations) provides fundamental platforms for understanding structure-activity relationships and rational catalyst design. Common synthesis approaches include (1) pyrolysis of metal–organic frameworks (MOFs) containing metal nodes and nitrogen-rich linkers; (2) high-temperature treatment of metal salts with nitrogen-containing precursors (melamine, cyanamide, polyaniline) and carbon supports; (3) atomic layer deposition of metal precursors followed by nitrogen incorporation; and (4) ball milling methods enabling mechanochemical synthesis. MOF-derived SACs offer advantages of uniform metal distribution and hierarchical porosity inherited from MOF templates [124].

State-of-the-art Fe-N-C catalysts achieve half-wave potentials of 0.80–0.85 V vs. RHE in alkaline media and 0.75–0.80 V in acidic media for ORR, approaching or matching commercial Pt/C performance in alkaline conditions [126]. The intrinsic activity of individual M-N_4_ sites has been demonstrated to rival or exceed Pt on a per-site basis. However, lower site density compared to nanoparticles and reduced stability, particularly in acidic environments, remain challenges for practical implementation. Advanced characterization techniques including aberration-corrected scanning transmission electron microscopy (AC-STEM), X-ray absorption spectroscopy (XAS), and Mössbauer spectroscopy enable identification and structural characterization of single-atom sites. Computational studies combining density functional theory (DFT) and microkinetic modeling have revealed that optimal ORR activity requires balanced binding energies for oxygen intermediates, with Fe-N_4_ sites approaching the volcano peak.

#### 6.2.2. SACs on MXene and MOF Supports

MXene and MOF supports offer unique advantages for SAC stabilization and performance enhancement. MXenes provide high electrical conductivity, abundant surface functional groups for metal anchoring, and tunable surface chemistry through termination control. MXene-supported SACs demonstrate superior charge transfer kinetics compared to carbon-supported analogs, with Co-N-MXene catalysts achieving onset potentials of 0.91 V vs. RHE for ORR [124]. MOF-derived carbons inherit porosity and nitrogen doping from parent MOFs while providing robust anchoring sites for single atoms. The pyrolysis conditions can be optimized to balance metal loading, site density, and structural integrity. MXene@MOF hybrids combine benefits of both materials, with SACs distributed throughout the hierarchical structure providing high atom utilization and facile mass transport [122,124]. SACs play emerging roles in battery chemistry beyond traditional electrocatalysis. In lithium-sulfur batteries, SACs anchored on conductive supports catalyze polysulfide conversion reactions, reducing shuttle effect and improving cycle life. In solid-state batteries, SACs at electrode–electrolyte interfaces facilitate charge transfer and reduce interfacial resistance. In metal–air batteries, bifunctional SACs catalyze both oxygen reduction during discharge and oxygen evolution during charge, enabling efficient round-trip cycling.

#### 6.2.3. Challenges and Design Principles

Single-atom catalysts face stability challenges including metal agglomeration, site poisoning, and support degradation. Strategies for stability enhancement include strong metal–support interactions through covalent bonding or coordination; confinement effects using porous supports; protective coatings; and defect engineering to create robust anchoring sites [124]. Achieving high metal loading (>5 wt%) while maintaining atomic dispersion represents a fundamental challenge. Excessive loading leads to clustering and nanoparticle formation. Hierarchical supports with multi-scale porosity and abundant anchoring sites enable higher loadings while preserving atomic dispersion. For multi-electron reactions such as CO_2_ reduction and nitrogen reduction, product selectivity depends critically on the electronic structure and coordination environment of single-atom sites. Computational screening combined with experimental validation has identified descriptor-based design principles, such as the d-band center model and binding energy scaling relationships, enabling rational catalyst design for targeted products [127].

### 6.3. High-Entropy Materials

High-entropy materials (HEMs) incorporating five or more elements in near-equimolar ratios represent a paradigm shift from conventional materials design focused on one or two principal elements. The high configurational entropy stabilizes single-phase solid solutions that would be immiscible in conventional synthesis, enabling access to unprecedented compositional space and emergent properties arising from synergistic multi-element interactions. 

#### 6.3.1. High-Entropy Alloys (HEAs)

High-entropy alloys demonstrate promising electrocatalytic activity for various reactions, with the multi-element composition providing numerous active site configurations and potentially synergistic effects. HEAs exhibit four core effects: cocktail effect—synergistic interactions among elements; lattice distortion—strain fields modifying electronic structure; sluggish diffusion—kinetic stabilization; and high entropy—thermodynamic stabilization of single-phase structures. HEA nanoparticles containing Pt, Pd, Rh, Ru, and Ir have demonstrated superior ORR activity and durability compared to commercial Pt/C, attributed to ensemble effects and electronic modifications from multi-element alloying. PGM-free HEAs such as FeCoNiMnCu exhibit bifunctional activity for ORR and OER, enabling application in rechargeable metal-air batteries. For HER, HEAs containing Mo, W, Co, Ni, and Fe achieve low overpotentials and Tafel slopes approaching those of Pt-based catalysts. HEAs demonstrate enhanced activity and CO tolerance for methanol and ethanol oxidation reactions in direct alcohol fuel cells. The multiple active site types in HEAs facilitate different reaction steps, potentially enabling more efficient multi-step reaction pathways compared to single-element catalysts [128].

#### 6.3.2. High-Entropy Layered Oxides (HELOs)

High-entropy layered oxides have emerged as a transformative strategy for stabilizing lithium-ion, sodium-ion, and potassium-ion battery cathodes. HELOs with the general formula LiM_1_M_2_M_3_M_4_M_5_O_2_ (where M represents transition metals such as Ni, Co, Mn, Fe, Ti, Cr, etc.) leverage configurational entropy to stabilize layered structures and suppress detrimental phase transitions during cycling. The high configurational entropy (ΔSconfig > 1.5R, where R is the gas constant) stabilizes the layered α-NaFeO_2_ structure and suppresses transformation to spinel or rock-salt phases that degrade capacity and rate capability. The diverse local coordination environments around Li^+^ sites create a “buffering effect” that accommodates structural strain during lithiation/delithiation, enhancing mechanical stability. HELOs demonstrate enhanced structural stability with minimal lattice parameter changes during cycling; suppressed voltage fade through mitigation of transition metal migration; improved rate capability from diverse ion diffusion pathways; and tunable redox activity through compositional optimization. Li(Ni_0.2_Co_0.2_Mn_0.2_Fe_0.2_Ti_0.2_)O_2_ exhibits reversible capacity exceeding 200 mAh g^−1^ with excellent capacity retention over 500 cycles [129]. Rational design of HELOs requires balancing multiple factors including electrochemical activity (Ni and Co provide high capacity; Mn provides structural stability); electronic conductivity (Co and Ni enhance conductivity); thermal stability (Ti, Cr, and Al improve stability); and cost and sustainability (Fe and Mn are abundant and inexpensive). Machine learning approaches combined with high-throughput computation enable accelerated exploration of the vast compositional space.

#### 6.3.3. High-Entropy Oxides (HEOs)

High-entropy oxides extend the HEM concept to oxide materials, offering applications in electrocatalysis and energy storage. HEOs such as (Co, Cr, Fe, Mn, Ni)_3_O_4_ with spinel structure and (Co, Cu, Mg, Ni, Zn)O with rock-salt structure exhibit single-phase stability despite containing five or more metal cations. HEO spinels demonstrate OER activity comparable to or exceeding benchmark RuO_2_ catalysts, with overpotentials of 280–320 mV at 10 mA cm^−2^ in alkaline media. The multiple metal cation types create diverse active sites, and synergistic electronic interactions optimize binding energies for oxygen intermediates. The structural stability imparted by configurational entropy enhances long-term durability compared to single or binary metal oxides [128]. HEOs serve as conversion-type anode materials for lithium-ion batteries, exhibiting capacities of 800–1200 mAh g^−1^. The multiple redox-active metals enable high capacity, while the entropy stabilization maintains structural integrity during repeated conversion reactions. HEOs also demonstrate pseudocapacitive behavior suitable for supercapacitor applications.

#### 6.3.4. Synthesis Challenges and Future Outlook

Achieving phase-pure HEMs with uniform elemental distribution requires careful control of synthesis conditions. Common methods include co-precipitation followed by calcination for HEOs, mechanical alloying or arc melting for HEAs, and sol–gel or hydrothermal synthesis for complex structures. Inhomogeneous elemental distribution and secondary phase formation remain common challenges [128,129]. The fundamental origins of enhanced properties in HEMs remain debated. Distinguishing contributions from configurational entropy, local distortion, and specific element synergies requires advanced characterization combining techniques such as synchrotron X-ray diffraction, extended X-ray absorption fine structure (EXAFS), and atomic-resolution electron microscopy with computational modeling. Promising research directions include medium-entropy materials (3–4 elements) balancing complexity and synthetic accessibility; high-entropy sulfides, phosphides, and nitrides for broader application scope; hierarchical structures combining HEM active phases with conductive scaffolds; computational screening using machine learning to identify optimal compositions; and operando characterization to understand dynamic structural evolution during electrochemical operation.

### 6.4. Metal–Organic Frameworks (MOFs) and Covalent Organic Frameworks (COFs)

Porous crystalline frameworks offer exceptional design flexibility, with tunable pore structures, high surface areas, and designable active sites enabling targeted optimization for diverse electrochemical applications. MOFs and COFs represent distinct yet complementary approaches to framework materials, with MOFs featuring metal–ligand coordination bonds and COFs featuring entirely covalent organic linkages.

#### 6.4.1. MOFs as Electrode Materials and Precursors

Electrically conductive MOFs incorporating redox-active metal centers and conjugated organic linkers serve as electrode materials for batteries and supercapacitors. Ni_3_(HITP)_2_ (HITP = 2,3,6,7,10,11-hexaiminotriphenylene) exhibits electrical conductivity exceeding 5000 S m^−1^ and demonstrates reversible lithium storage with capacity of 90 mAh g^−1^. Conductive MOFs also show promise for electrocatalysis, with Fe-based MOFs demonstrating ORR activity in fuel cells [122]. Pyrolysis of MOFs under inert atmosphere produces porous carbons, metal/carbon composites, or metal oxide/carbon hybrids inheriting hierarchical porosity from MOF templates while gaining electrical conductivity from carbonization. MOF-derived carbons exhibit high surface areas (1000–3000 m^2^ g^−1^), tunable pore size distributions, and heteroatom doping (N, S, P) from organic linkers, providing excellent performance as supercapacitor electrodes and battery anodes. Integration of MXenes with MOFs creates synergistic composites combining high conductivity and mechanical stability of MXenes with high surface area and tailored porosity of MOFs [122,123]. In situ growth of MOF crystals on MXene sheets prevents MXene restacking while providing abundant active sites. MXene@ZIF-67-derived composites achieve specific capacitances exceeding 1000 F g^−1^ for supercapacitors and demonstrate enhanced lithium-ion battery performance compared to individual components.

#### 6.4.2. COFs for Sustainable Energy Storage

Covalent organic frameworks feature entirely covalent bonding between organic building blocks, offering advantages of high chemical stability, tunable porosity, and incorporation of organic redox-active moieties. COFs provide sustainable alternatives to inorganic electrode materials, with potential for recyclability and reduced environmental impact. COFs are constructed from organic building blocks connected through covalent bonds such as boronate ester, imine, hydrazone, or triazine linkages. The crystalline nature enables precise structural control, with pore sizes tunable from microporous (<2 nm) to mesoporous (2–50 nm) regimes. Conjugated COF backbones enhance electrical conductivity, while redox-active functional groups provide charge storage capacity. COFs serve as cathode materials for lithium-ion and sodium-ion batteries through organic redox reactions involving quinone, imine, azo, or carbonyl groups. Anthraquinone-based COFs achieve specific capacities of 150–200 mAh g^−1^ with excellent rate capability and long cycle life exceeding 1000 cycles. The flexible framework accommodates ion insertion without significant structural degradation, addressing the volume change challenges of inorganic electrode materials.

The limited electrical conductivity of most COFs (10^−10^ to 10^−6^ S m^−1^) necessitates strategies such as conjugated backbone design incorporating aromatic building blocks and planar structures promoting π-π stacking; heteroatom doping introducing nitrogen, sulfur, or boron; composite formation with conductive additives (carbon nanotubes, graphene, MXenes); and guest molecule intercalation. Recent advances in fully conjugated COFs have achieved conductivities exceeding 1 S m^−1^ [131,132,133,134,135,136,137,138,139,140,141]. COFs offer advantages of sustainability (earth-abundant elements), tunable redox potentials through molecular design, and structural diversity. Challenges include limited electrical conductivity, lower volumetric energy density compared to inorganic materials, and sensitivity to moisture for certain linkage chemistries. Future research directions emphasize development of conductive COF backbones, integration with inorganic components in hybrid electrodes, and exploration of COF-derived carbons combining porosity with conductivity.

### 6.5. Fabrication Strategies and Processing Techniques

#### 6.5.1. Three-Dimensional Printing and Additive Manufacturing

Direct ink writing (DIW) enables fabrication of electrodes with complex 3D geometries, controlled porosity, and functionally graded compositions. Inks containing active materials, conductive additives, binders, and rheology modifiers are extruded through fine nozzles to build structures layer by layer. DIW has been applied to fabricate battery electrodes with interdigitated architectures, supercapacitor electrodes with hierarchical porosity, and fuel cell electrodes with optimized gas transport channels. The ability to create high-aspect-ratio features and integrate multiple materials within single structures provides unprecedented design flexibility [132]. Selective laser sintering (SLS) uses laser energy to fuse powder particles into solid structures, enabling fabrication of metal and ceramic electrodes with complex geometries. Stereolithography (SLA) employs photopolymerizable resins containing active material precursors, with UV light selectively curing regions to build structures layer by layer. Post-processing steps including de-binding and sintering convert polymer-derived structures into functional electrodes. These techniques enable rapid prototyping and customization of electrode architectures for specific applications. 3D printing offers advantages of design freedom, rapid prototyping, and potential for integrated device manufacturing. Challenges include limited material selection (inks must satisfy rheological requirements), relatively slow fabrication speeds for large-scale production, and achieving high electrical conductivity and electrochemical performance comparable to conventional electrodes. Future developments emphasize multi-material printing, in situ monitoring and control, and scaling toward industrial production.

#### 6.5.2. Electrospinning and Nanofiber Technologies

Electrospinning produces continuous nanofibers with diameters ranging from tens of nanometers to micrometers by applying high voltage to polymer solutions, creating high-surface-area electrode structures with interconnected porosity. Electrospun nanofibers can incorporate various materials including polymers, carbons, metal oxides, and composites. The one-dimensional structure provides direct electron transport pathways and accommodates volume changes in battery electrodes, enhancing rate capability and cycle life [133]. Coaxial electrospinning enables fabrication of core–shell nanofibers with distinct core and shell compositions, providing opportunities for functional design. For example, conductive carbon core with active material shell combines efficient electron transport with high active material loading. Electrospun nanofibers can be carbonized to produce carbon nanofibers with high surface area and electrical conductivity or serve as templates for metal oxide nanotubes after core removal. Electrospun nanofiber electrodes have been demonstrated for lithium-ion batteries (silicon/carbon composite nanofibers achieving > 1000 mAh g^−1^), sodium-ion batteries (hard carbon nanofibers with 250–300 mAh g^−1^), supercapacitors (heteroatom-doped carbon nanofibers with 200–300 F g^−1^), fuel cells (PGM-free ORR catalysts supported on nanofibers), and sensors (high-surface-area sensing electrodes).

#### 6.5.3. Atomic Layer Deposition (ALD)

ALD provides atomic-level control over film thickness and composition through sequential, self-limiting surface reactions, enabling conformal coating of complex three-dimensional structures. ALD has been employed to create ultrathin catalyst layers (1–10 nm) maximizing catalyst utilization, protective coatings preventing electrode degradation, and precisely controlled core–shell structures optimizing performance. The low deposition temperatures (typically 100–300 °C) preserve underlying materials, while the self-limiting surface reactions ensure uniformity across high-aspect-ratio features. ALD-deposited Al_2_O_3_, ZrO_2_, or TiO_2_ coatings (1–5 nm thickness) on high-nickel layered oxide cathodes suppress surface degradation and improve cycle life in lithium-ion batteries. ALD-synthesized Pt catalysts with atomic layer thickness achieve high mass activity for fuel cell ORR. ALD also enables synthesis of complex materials including ternary oxides and doped materials with precise compositional control [122].

#### 6.5.4. Plasma Processing

Plasma processing methods, including plasma-enhanced chemical vapor deposition (PECVD), plasma etching, and plasma treatment, enable surface modification and nanostructure creation. Plasma techniques can introduce functional groups (oxygen, nitrogen, fluorine), create defects and active sites, deposit thin films, and etch materials with high precision. The non-equilibrium nature of plasma processes enables low-temperature synthesis of materials and surface modifications difficult to achieve through thermal processes [119]. Plasma treatment of carbon electrodes introduces oxygen and nitrogen functional groups, enhancing wettability and pseudocapacitance for supercapacitors. Plasma-enhanced CVD enables low-temperature synthesis of graphene and carbon nanotubes on temperature-sensitive substrates. Plasma etching creates hierarchical pore structures and increases surface area. Nitrogen plasma treatment of MXenes modifies surface terminations and introduces nitrogen doping, enhancing catalytic activity.

#### 6.5.5. In Situ Growth and Self-Assembly

Hydrothermal and solvothermal methods enable in situ growth of active materials directly on conductive substrates (nickel foam, carbon cloth, current collectors), creating binder-free electrodes with strong interfacial adhesion and efficient charge transport. The solution-based synthesis occurs in sealed autoclaves at elevated temperatures (100–250 °C), enabling crystallization of metal oxides, hydroxides, sulfides, and other materials with controlled morphologies (nanowires, nanosheets, nanoflowers). The direct growth on substrates eliminates need for binders and conductive additives, increasing active material content and reducing interfacial resistance [132]. Self-assembly processes driven by electrostatic interactions, hydrogen bonding, or π-π stacking enable fabrication of hierarchical structures with controlled organization at multiple length scales. Layer-by-layer assembly builds composite films with alternating layers of different materials, enabling precise control over composition and thickness. Self-assembly of MXene nanosheets with MOF nanocrystals creates hybrid structures with intimate interfacial contact and synergistic properties [122,123]. Template methods using sacrificial materials (silica spheres, polystyrene beads, metal–organic frameworks, bacterial cellulose) enable fabrication of structured electrodes with controlled morphology and porosity. After synthesis of active materials around or within templates, template removal through etching, calcination, or dissolution leaves behind desired structures. Inverse opal structures, hollow spheres, and hierarchical porous architectures accessible through template methods provide high surface area and optimized mass transport [132]. Table 1 compares the performance of different electrode materials.

## 7. Challenges and Future Perspectives

Despite significant progress in electrode material development, numerous challenges remain for advancing electrochemical devices toward widespread deployment and optimal performance. Many electrochemical processes remain incompletely understood at the molecular level. The complexity of electrode–electrolyte interfaces, the multitude of possible reaction intermediates, and the challenges of characterizing buried interfaces under operating conditions limit rational catalyst and electrode design. Advanced in situ and operando characterization techniques, including synchrotron-based spectroscopies, environmental electron microscopy, and scanning probe microscopies, provide unprecedented insights into electrochemical processes. Complementary computational approaches, including density functional theory calculations and molecular dynamics simulations, enable prediction of material properties and reaction mechanisms. Integration of experimental and computational studies through iterative feedback loops promises to accelerate material discovery and optimization. Long-term stability represents a critical requirement for practical electrochemical devices, yet many promising electrode materials suffer from degradation during operation. Mechanisms of degradation include dissolution, corrosion, structural changes, active site poisoning, and loss of electrical connectivity. Understanding degradation mechanisms and developing mitigation strategies require systematic studies under realistic operating conditions. Strategies for improving stability include protective coatings, structural engineering for mechanical robustness, compositional optimization for chemical stability, and development of self-healing materials.

Laboratory-scale synthesis methods often cannot be directly translated to industrial production, creating gaps between research achievements and commercial implementation. Scalable synthesis approaches that maintain material quality while reducing cost and environmental impact are essential. Continuous flow synthesis, mechanochemical synthesis, and other scalable techniques show promise for bridging the laboratory-to-industry gap. Additionally, electrode fabrication processes must be compatible with high-throughput manufacturing while achieving necessary performance characteristics. The environmental impact of electrode materials throughout their lifecycle—from raw material extraction through manufacturing, use, and end-of-life disposal—requires careful consideration. Sustainable materials design should prioritize abundant, non-toxic elements, minimize energy-intensive processing, and enable recycling and recovery. Development of biodegradable or environmentally benign electrode materials, particularly for single-use applications such as certain sensors, represents an important direction. Design for recyclability, including ease of disassembly and material separation, should be incorporated from the outset of device development. Future electrode materials may incorporate multiple functionalities beyond electrochemical activity, including self-healing capability, environmental responsiveness, or integrated sensing. Smart materials that adapt their properties based on operating conditions could optimize performance across varying demands. Hierarchical structures combining multiple length scales and material types may provide synergistic benefits exceeding those of individual components. The vast compositional and structural space of possible electrode materials exceeds the capacity for exhaustive experimental exploration. Machine learning approaches, including neural networks, genetic algorithms, and Bayesian optimization, enable accelerated materials discovery through prediction of material properties and guided experimental design. High-throughput experimental platforms combined with automated characterization and machine learning analysis create feedback loops for rapid materials optimization. However, the quality and quantity of training data, the interpretability of models, and the validation of predictions remain important considerations. Electrode performance cannot be considered in isolation from other device components, including electrolytes, separators, current collectors, and device architectures. System-level optimization considering interactions between components and trade-offs between competing performance metrics (energy density versus power density, activity versus stability, cost versus performance) requires holistic approaches. Multiphysics modeling integrating electrochemical, thermal, and mechanical phenomena enables device-level optimization and identification of performance-limiting factors.

## 8. Conclusions

Electrode materials represent the foundation of electrochemical devices, determining their performance, efficiency, stability, and cost. The diverse range of applications—from energy storage and conversion to sensing and chemical production—requires correspondingly diverse material properties and functionalities. Traditional electrode materials, while enabling current technologies, face limitations that emerging materials aim to address through novel compositions, structures, and properties. Significant progress has been achieved in developing advanced electrode materials, including nanostructured materials with enhanced surface area and reaction kinetics, multi-component materials exhibiting synergistic effects, two-dimensional materials offering unique properties, and single-atom catalysts maximizing metal utilization. These advances have enabled improvements in battery energy density, fuel cell efficiency, electrolysis performance, and sensor sensitivity.

Despite these advances, several critical challenges remain, including incomplete fundamental understanding of electrochemical processes, stability limitations, scalability gaps between laboratory and industry, and sustainability concerns. Many high-performance electrode materials suffer from insufficient long-term stability under realistic operating conditions, including dissolution, structural reconstruction, catalyst poisoning, and mechanical degradation. In addition, the scalability of laboratory-scale synthesis methods, compatibility with industrial fabrication processes, and environmental sustainability of material choices continue to limit practical deployment. Addressing these challenges requires deeper fundamental understanding of electrode–electrolyte interfaces, degradation mechanisms, and structure–activity relationships under operando conditions.

Future research should emphasize rational materials design guided by in situ/operando characterization and computational modeling, coupled with scalable and environmentally benign synthesis strategies. The integration of machine learning and data-driven approaches offers powerful opportunities to accelerate materials discovery and optimize multi-parameter performance trade-offs. The future of electrode materials lies in rational design based on fundamental understanding, sustainable materials choices considering full lifecycle impacts, and multifunctional materials providing integrated capabilities. Moreover, system-level optimization—considering electrodes in conjunction with electrolytes, membranes, current collectors, and device architectures—will be essential for translating material-level advances into commercial technologies. Overall, continued innovation in electrode materials, guided by sustainability and manufacturability considerations, will be central to enabling next-generation electrochemical devices that support global energy transition and environmental stewardship.

## Figures and Tables

**Table 1:** Performance of different electrode materials.

No.	Material/Electrode Type	Synthesis/Structure	Device Type	Specific Capacitance/Capacity	Energy Density	Cycle Stability	Reference
1	LiCoO_2_ (SCC)	Sol–gel, irregular grains	LIB cathode	206.1 mAh g^−1^ @ 0.1C	760 Wh kg^−1^ (calc.)	81.8% after 51 cycles	[29]
2	Al, La-doped LiCoO_2_	Solid-state	LIB cathode	224.9 mAh g^−1^ @ 0.1C	820 Wh kg^−1^	78% after 200 cycles	[29]
3	LiNi_0.5_Mn_1.5_O_4_	Co-precipitation, octahedron	LIB cathode	125 mAh g^−1^ @ 0.5C	575 Wh kg^−1^	98.3% after 100 cycles	[29]
4	Fe_3_O_4_–α-Fe_2_O_3_	Hybrid oxide	LIB anode	1100 mAh g^−1^ @ 0.1 A g^−1^	700 Wh kg^−1^	97% after 40 cycles	[40]
5	Cu_3_SnS_4_	Sulfide nanostructure	LIB anode	1082 mAh g^−1^ @ 0.2 A g^−1^	690 Wh kg^−1^	76% after 950 cycles	[54]
6	Fe_3_SnC@CNF	Carbide-carbon nanofiber	LIB anode	600 mAh g^−1^ @ 0.1 A g^−1^	410 Wh kg^−1^	96% after 1000 cycles	[76]
7	Ti_3_C_2_Tx MXene	Etched MAX phase	Supercapacitor	330 F g^−1^ @ 1 A g^−1^	120 Wh kg^−1^	95% after 1000 cycles	[92]
11	Ni–P	Electroless plating	Supercapacitor/AWS	159 mF cm^−2^ @ 10 mA cm^−2^	≈110 µWh cm^−2^	100 h stable	[78]
12	Ni–P–B	Electroless plating	Supercapacitor	76 mF cm^−2^ @ 10 mA cm^−2^	263 µWh cm^−2^	240 h @ 1000 mA cm^−2^	[79]
13	Co–Ni–P	Electroless plating	Supercapacitor	125 mF cm^−2^ @ 10 mA cm^−2^	320 µWh cm^−2^	24 h stable	[76]

## Data Availability

No new data was created or analyzed in this study.

## References

[1] Ambrosi A., Moo J.G.S., Pumera M. (2016). Helical 3D-printed metal electrodes as custom-shaped 3D platform for electrochemical devices. Adv. Funct. Mater..

[2] Jerkiewicz G. (2022). Applicability of platinum as a counter-electrode material in electrocatalysis research. ACS Catal..

[3] Kuang Y., Chen C., Kirsch D., Hu L. (2019). Thick electrode batteries: Principles, opportunities, and challenges. Adv. Energy Mater..

[4] Majlan E.H., Rohendi D., Daud W.R.W., Husaini T., Haque M.A. (2018). Electrode for proton exchange membrane fuel cells: A review. Renew. Sustain. Energy Rev..

[5] Suffredini H.B., Cerne J.L., Crnkovic F.C., Machado S.A.S., Avaca L.A. (2000). Recent developments in electrode materials for water electrolysis. Int. J. Hydrogen Energy.

[6] Sun J., Wu C., Sun X., Hu H., Zhi C., Houa L., Yuan C. (2017). Recent progresses in high-energy-density all pseudocapacitive-electrode-materials-based asymmetric supercapacitors. J. Mater. Chem. A.

[7] Sumdani M.G., Islam M.R., Yahaya A.N.A., Safie S.I. (2022). Recent advancements in synthesis, properties, and applications of conductive polymers for electrochemical energy storage devices: A review. Polym. Eng. Sci..

[8] Su Y.-F., Wu F., Bao L.-Y., Yang Z.-H. (2007). RuO_2_/activated carbon composites as a positive electrode in an alkaline electrochemical capacitor. New Carbon Mater..

[9] Liu Z., Qin L., Cao X., Zhou J., Pan A., Fang G., Wang S., Liang S. (2022). Ion migration and defect effect of electrode materials in multivalent-ion batteries. Prog. Mater. Sci..

[10] Chen S., Qiu L., Cheng H.-M. (2020). Carbon-based fibers for advanced electrochemical energy storage devices. Chem. Rev..

[11] Batista C.Y.P., Romaguera-Barcelay Y., Matos R.S., Pedraça A.D.S.A., Amâncio M.D.A., Kourouma A., Ruiz Y.L., Cotta E.A., Brito W.R., Gandarilla A.M.D. (2023). Morphology, microstructure, and electrocatalytical properties of sol-gel spin-coated Bi_0.5_Na_0.5_Ba(TiO_3_)_2_ thin films. Appl. Surf. Sci..

[12] Karnati R.K., Bakir E.M. (2023). Smart and reusable electrochemical sensor based on Ag@SiO_2_ gel for the detection of sulfur-based compounds in environmental samples. J. Sol-Gel Sci. Technol..

[13] Zhang X., Lu X., Jia X., Liu H., Niu Y. (2024). Design and fabrication of ellipsoidal α-Fe_2_O_3_@SnO_2_ core–shell microspheres with heterostructures for improving photocatalytic performance. Inorg. Chem. Commun..

[14] Liu Z., Shi W., Lei Y., Xie Z. (2023). Novel polyamide/silica/chitosan covalent hybrid: One-step BIC/sol-gel preparation at room temperature and dual applications in Hg2+ electrochemical probing and dye adsorption. Carbohydr. Polym..

[15] Noreen S., Tahir M.B., Hussain A., Nawaz T., Rehman J.U., Dahshan A., Alzaid M., Alrobei H. (2022). Emerging 2D-nanostructured materials for electrochemical and sensing application—A review. Int. J. Hydrogen Energy.

[16] Ren D., Xie L., Wang L., He X. (2021). A practical approach to predict volume deformation of lithium-ion batteries from crystal structure changes of electrode materials. Int. J. Energy Res..

[17] Xu W., Zhao X., Zhan F., He Q., Wang H., Chen J., Wang H., Ren X., Chen L. (2022). Toward emerging two-dimensional nickel-based materials for electrochemical energy storage: Progress and perspectives. Energy Storage Mater..

[18] Verma D., Dubey N., Yadav A.K., Saraya A., Sharma R., Solanki P.R. (2024). Disposable paper-based screen-printed electrochemical immunoplatform for dual detection of esophageal cancer biomarkers in patients’ serum samples. Mater. Adv..

[19] Tu J.X., Chen X.X., Xiong X.B., Chen Y., Ma J., Cao H.Y., Li A.J. (2023). Microwave hydrothermal electrodeposition of nickel carbonate hydroxide/cobalt hydroxide film on nickel foam for cement-based structural supercapacitors. Mater. Today Chem..

[20] Mouraliraman D., Thiagarajan A., Deepa S., Sriram G., Aruchamy K., Oh T.H., Shin D. (2024). 3D printed lithium-ion batteries: An in-depth examination of the advancements in flexibility and stand-alone capability. J. Energy Storage.

[21] Mathankumar M., Balasubramanian S., Hasin P., Lin J.-Y. (2024). Pulsed laser deposition as an efficient tool to enhance the performance of electrocatalysis design, strategies and current perspectives. Int. J. Hydrogen Energy.

[22] Joshi B., Samuel E., Kim Y.-I., Yarin A.L., Swihart M.T., Yoon S.S. (2021). Electrostatically sprayed nanostructured electrodes for energy conversion and storage devices. Adv. Funct. Mater..

[23] Jang I., Hankin A., Xie Z., Skinner S.J., Kelsall G.H. (2024). Structural effects of 3D inkjet-printed Ni(O)-YSZ pillared electrodes on performances of solid oxide electrochemical reactors. Small.

[24] Hao F., Verma A., Mukherjee P.P. (2019). Electrodeposition stability of metal electrodes. Energy Storage Mater..

[25] Haldorai Y., Kumar R.S., Ramesh S., Kumar R.T.R., Yang W. (2024). Chemical vapor deposition-grown single-layer graphene-supported nanostructured Co_3_O_4_ composite as binder-free electrode for asymmetric supercapacitor and electrochemical detection of caffeic acid. J. Alloys Compd..

[26] Haider A.J., Rsool R.A., Haider M.J., Rsool R.A., Dheyab A.B. (2020). Properties of LiCo0.5Ni0.45Ag0.05O2 thin films for high storage energy capacity by pulsed laser deposition. Energy Rep..

[27] Guo J., Pan L., Sun J., Wei D., Dai Q., Xu J., Li Q., Han M., Wei L., Zhao T. (2024). Metal-free fabrication of nitrogen-doped vertical graphene on graphite felt electrodes with enhanced reaction kinetics and mass transport for high-performance redox flow batteries. Adv. Energy Mater..

[28] Gleason K.K. (2024). Designing organic and hybrid surfaces and devices with initiated chemical vapor deposition (iCVD). Adv. Mater..

[29] Wang Y., Wang E., Zhang X., Yu H. (2021). High-Voltage “ Single-Crystal ” Cathode Materials for Lithium-Ion Batteries. Energy Fuels.

[30] George A., Kundu M. (2023). Tailoring the surface morphology of a nanostructured CuCo_2_S_4_ electrode by surfactant-assisted electrodeposition for asymmetric supercapacitors with high energy and power density. Energy Fuel.

[31] Garakani M.A., Bellani S., Pellegrini V., Oropesa-Nuñez R., Castillo A.E.D.R., Abouali S., Najafi L., Martín-García B., Ansaldo A., Bondavalli P. (2021). Scalable spray-coated graphene-based electrodes for high-power electrochemical double-layer capacitors operating over a wide range of temperature. Energy Storage Mater..

[32] Chen K.-Y., Biswas A., Cai S., Huang J., Andrews J. (2024). Inkjet printed potentiometric sensors for nitrate detection directly in soil enabled by a hydrophilic passivation layer. Adv. Mater. Technol..

[33] Chang C.-M., Chiang Y.-C., Cheng M.-H., Lin S.-H., Jian W.-B., Chen J.-T., Cheng Y.-J., Ma Y.-R., Tsukagoshi K. (2021). Fabrication of WO_3_ electrochromic devices using electro-exploding wire techniques and spray coating. Sol. Energy Mater. Sol. Cells.

[34] Ambaye A.D., Kefeni K.K., Mishra S.B., Nxumalo E.N., Ntsendwana B. (2021). Recent developments in nanotechnology-based printing electrode systems for electrochemical sensors. Talanta.

[35] Alahmad W., Cetinkaya A., Kaya S.I., Varanusupakul P., Ozkan S.A. (2023). Electrochemical paper-based analytical devices for environmental analysis: Current trends and perspectives. Trends Environ. Anal. Chem..

[36] Ding Z.-J., Liu Y., Weerasooriya R., Chen X. (2024). Electrochemical determination of chromium (VI) with Au/UiO-66 modified glassy carbon and screen-printed electrodes by linear sweep voltammetry (LSV). Anal. Lett..

[37] Dobrovodsky D., Danhel A., Renciuk D., Mergny J.-L., Fojta M. (2024). N-methyl mesoporphyrin IX (NMM) as electrochemical probe for detection of guanine quadruplexes. Bioelectrochemistry.

[38] Du J., Wang W., Wan M., Wang X., Li G., Tan Y., Li C., Tu S., Sun Y. (2021). Doctor-blade casting fabrication of ultrathin Li metal electrode for high-energy-density batteries. Adv. Energy Mater..

[39] Honeychurch K.C. (2017). Cheap and disposable gold and silver electrodes: Trends in the application of compact discs and digital versatile discs for electroanalytical chemistry. TrAC Trends Anal. Chem..

[40] Roy K., Banerjee A., Ogale S. (2022). Search for New Anode Materials for High Performance Li-Ion Batteries. ACS Appl. Mater. Interfaces.

[41] Li Y., Han H., Fei Y., Pan D., Wang H. (2024). A honeycomb-like micro-needle sensor with gold nanoparticles embedded for the determination of hexavalent chromium in seawater. Microchem. J..

[42] Liu Y., Zheng Y., Ren Y., Wang Y., You S., Liu M. (2024). Selective nitrate electroreduction to NH3on CNT electrodes with controllable interfacial wettability. Environ. Sci. Technol..

[43] Mounesh, Reddy K.R.V., Pandith A., Eldesoky G.E., Nagaraja B.M. (2024). Novel nitrogen-rich anchored nickel (II) phthalocyanine with composite of multiwalled carbon nanotubes on modified glassy carbon electrode: Sensitive and selective electrocatalytic activity of nitrite. Appl. Organomet. Chem..

[44] Wahyuni W.T., Putra B.R., Rahman H.A., Anindya W., Hardi J., Rustami E., Ahmad S.N. (2024). Electrochemical sensors based on gold–silver core–shell nanoparticles combined with a Graphene/PEDOT:PSS composite modified glassy carbon electrode for paraoxon-ethyl detection. ACS Omega.

[45] Wu Y., He N., Liang G., Zhang C., Liang C., Ho D., Wu M., Hu H. (2024). Thick-network electrode: Enabling dual working voltage plateaus of Zn-ion micro-battery with ultra-high areal capacity. Adv. Funct. Mater..

[46] Zhang H., Wang Q., Li L., Huang R., Gu H., Chen H., Wu Z., Wang Z. (2024). Electric double layer capacitive adsorption and faradaic pseudo-capacitance behavior of ZnFe-PANI/CNT electrode for phosphate removal in capacitive deionization. Sep. Purif. Technol..

[47] Xie M., Yao G., Gan X., Zhang C., Zhang T., Wang Q., Li X., Zhou C., Zhao K., Gao M. (2024). Non-enzyme, temperature calibrating, and bioactive fiber-based flexible sensors for dopamine and lactic acid detection. Adv. Fiber Mater..

[48] Wang X., Hao L., Du R., Wang H., Dong J., Zhang Y. (2024). Synthesis of unique three-dimensional CoMn_2_O_4_@Ni(OH)_2_ nanocages via Kirkendall effect for non-enzymatic glucose sensing. J. Colloid Interface Sci..

[49] Shao W., Yan R., Zhou M., Ma L., Roth C., Ma T., Cao S., Cheng C., Yin B., Li S. (2023). Carbon-based electrodes for advanced zinc-air batteries: Oxygen-catalytic site regulation and nanostructure design. Electrochem. Energy Rev..

[50] Miao Q., Wu K., Sheng L., Shi H., Jiang L., Le L., Fan Z. (2024). All-functionalized-graphene ribbon films for flexible asymmetric supercapacitors with ultrahigh energy and power densities. Chem. Eng. J..

[51] Kumar Y.A., Alagarasan J.K., Ramachandran T., Rezeq M., Bajaber M.A., Alalwiat A.A., Moniruzzaman M., Lee M. (2024). The landscape of energy storage: Insights into carbon electrode materials and future directions. J. Energy Storage.

[52] Kothandam G., Singh G., Guan X., Lee J.M., Ramadass K., Joseph S., Benzigar M., Karakoti A., Yi J., Kumar P. (2023). Recent advances in carbon-based electrodes for energy storage and conversion. Adv. Sci..

[53] Khan M., Inamuddin (2024). Fabrication and characterization of electrically conducting electrochemically synthesized polypyrrole-based enzymatic biofuel cell anode with biocompatible redox mediator vitamin K3. Sci. Rep..

[54] Hwang I., Kim D., Choi J.W., Yoo D. (2024). Toward Practical Multivalent Ion Batteries with Quinone-Based Organic Cathodes. ACS Appl. Mater. Interfaces.

[55] Kangmennaa A., Forkuo R.B., Agorku E.S. (2024). Carbon-based electrode materials for sensor application: A review. Sens. Technol..

[56] Cang Y., Yuan Y., Zhang K., Li X.L., Song T., Xie J. (2024). Encapsulation of glucose oxidase on zeolitic imidazolate framework-67 collaborates with carbon nanotubes to enhance the electrochemical performance of the enzymatic electrode. Energy Fuel.

[57] Ahmad F., Zahid M., Jamil H., Khan M.A., Atiq S., Bibi M., Shahbaz K., Adnan M., Danish M., Rasheed F. (2023). Advances in graphene-based electrode materials for high-performance supercapacitors: A review. J. Energy Storage.

[58] Bibi F., Gerard O., Khan A.J., Khalid M., Numan A. (2024). Categories of pseudocapacitor: Intrinsic, extrinsic, and intercalation materials. Supercapacitors.

[59] Delbari S.A., Ghadimi L.S., Hadi R., Farhoudian S., Nedaei M., Babapoor A., Namini A.S., Le Q.V., Shokouhimehr M., Asl M.S. (2021). Transition metal oxide-based electrode materials for flexible supercapacitors: A review. J. Alloys Compd..

[60] Du H., Wang Y., Kang Y., Zhao Y., Tian Y., Wang X., Tan Y., Liang Z., Wozny J., Li T. (2024). Side reactions/changes in lithium-ion batteries: Mechanisms and strategies for creating safer and better batteries. Adv. Mater..

[61] Groher C., Cupid D.M., Mautner A., Rosenberg E., Kahr J. (2024). Operando GC/MS for the investigation of different decomposition pathways during solid electrolyte interphase (SEI) formation with SEI forming additives. J. Power Sources.

[62] Kim J.H., Kim S., Kang Y.C., Pol V.G. (2024). Innovative amorphous multiple anionic transition metal compound electrode for extreme environments (≤−80 °C) battery operations. Nano Energy.

[63] Kim M., Park J., Yeo G., Ko M., Jang H. (2023). Designing CNT-implanted graphite felt as a sustainable electron network for long-cycling of vanadium redox flow batteries. Carbon.

[64] Li L., Nam J.S., Kim M.S., Wang Y., Jiang S., Hou H., Kim I.-D. (2023). Sulfur–carbon electrode with PEO-LiFSI-PVDF composite coating for high-rate and long-life lithium–sulfur batteries. Adv. Energy Mater..

[65] Parveen N. (2024). Resent development of binder-free electrodes of transition metal oxides and nanohybrids for high performance supercapacitors—A review. Chem. Rec..

[66] Rangaraj V.M., Yoo J.-I., Song J.-K., Mittal V. (2023). MOF-derived 3D MnO_2_@graphene/CNT Ag@graphene/CNT hybrid electrode materials for dual-ion selective pseudocapacitive deionization. Desalination.

[67] Sun C., Han Z., Wang X., Liu B., Li Q., Li H., Xu J., Cao J.-M., Wu X.-L. (2023). Advanced carbons nanofibers-based electrodes for flexible energy storage devices. Adv. Funct. Mater..

[68] Wang Z., Hong P., Zhao H., Lei Y. (2023). Recent developments and future prospects of transition metal compounds as electrode materials for potassium-ion hybrid capacitors. Adv. Mater. Technol..

[69] Yu T., Chen H., Hu T., Feng J., Xing W., Tang L., Tang W. (2024). Recent advances in the applications of encapsulated transition-metal nanoparticles in advanced oxidation processes for degradation of organic pollutants: A critical review. Appl. Catal. B.

[70] Yuan Z., Ma Y., Zhang P., Zhai M., Qin C., Jiang X. (2023). N-, P-, and Ni-Co-doped porous carbon from poplar powder and graphene oxide composites as electrode materials for supercapacitors. Energy Fuel.

[71] Zhang H., Tian J., Cui X., Li J., Zhu Z. (2023). Highly mesoporous carbon nanofiber electrodes with ultrahigh specific surface area for efficient capacitive deionization. Carbon.

[72] Zhu Z., Luo L., He Y., Mushtaq M., Li J., Yang H., Khanam Z., Qu J., Wang Z., Balogun M.-S. (2024). High-performance alkaline freshwater and seawater hydrogen catalysis by sword-head structured Mo_2_N-Ni_3_Mo_3_N tunable interstitial compound electrocatalysts. Adv. Funct. Mater..

[73] Zheng Y., Xiao S., Xing Z., Wu H., Ma T., Zeng Z., Liao Y., Li S., Cheng C., Zhao C. (2024). Oxophilic vanadium and deprotonated ruthenium atoms on tungsten carbide with accelerated intermediate migration for high-performance seawater hydrogen evolution. Nano Energy.

[74] Zhang R., Cao J., Peng T., Wu K., Shu Y. (2024). Flower-like NiCo_2_O_4_ spinel microspheres for efficient chlorine evolution under neutral conditions. Electrochim. Acta.

[75] Xie Y., Naguib M., Mochalin V.N., Barsoum M.W., Gogotsi Y., Yu X., Nam K.W., Yang X.Q., Kolesnikov A.I., Kent P.R.C. (2014). Role of surface structure on Li-Ion energy storage capacity of two-dimensional transition-metal carbides. J. Am. Chem. Soc..

[76] Ge Z., Xu S., Fu X., Zhao Z. (2025). Improving the Cold-Start Performance of Proton Exchange Membrane Fuel Cells via Precision Engineering of Key Materials. Precis. Chem..

[77] Vinothkumar V., Sekhar Y.C., Chen S.-M., Prasad G.V., Kim T.H. (2024). Fabrication of spinel MCr_2_O_4_(M = Ni and Co) nanostructures as positive electrode materials for high-performance supercapacitors. J. Energy Storage.

[78] Raza S., Hayat A., Bashir T., Chen C., Shen L., Orooji Y., Lin H. (2024). Electrochemistry of 2D-materials for the remediation of environmental pollutants and alternative energy storage/conversion materials and devices, a comprehensive review. Sustain. Mater. Technol..

[79] Oh Y., Theerthagiri J., Min A., Moon C.J., Yu Y., Choi M.Y. (2024). Pulsed laser interference patterning of transition-metal carbides for stable alkaline water electrolysis kinetics. Carbon Energy.

[80] Malone N., Fiedler H., Mitchell D.R.G., Kennedy J.V., Waterhouse G., Gupta P. (2024). Grain boundary-rich tungsten carbide nanoparticle films exhibit high intrinsic activity toward hydrogen evolution. ACS Appl. Nano Mater..

[81] Ma L., Jiang Y.-K., Xu D.-R., Fang Y.-Y., Li N., Cao D.-Y., Chen L., Lu Y., Huang Q., Su Y.-F. (2024). Enabling stable and low-strain lithium plating/stripping with 2D layered transition metal carbides by forming Li-zipped MXenes and a Li halide-rich solid electrolyte interphase. Angew. Chem. Int. Ed..

[82] Liu J., Yu L., Ran Q., Chen X., Wang X., He X., Jin H., Chen T., Chen J.S., Guo D. (2024). Regulating electron filling and orbital occupancy of anti-bonding states of transition metal nitride heterojunction for high areal capacity lithium–sulfur full batteries. Small.

[83] Hussain A.A., Rana A.K. (2022). Emerging 2D nanomaterial composites for efficient energy conversion: Insight into the evolutionary perspective of devices. 2D Nanomaterials for Energy and Environmental Sustainability, Materials Horizons: From Nature to Nanomaterials.

[84] Hu H., Wang X., Attfield J.P., Yang M. (2024). Metal nitrides for seawater electrolysis. Chem. Soc. Rev..

[85] Bustos E.B. (2024). High-performance transition metal oxide electrodes for water treatment. Curr. Opin. Electrochem..

[86] Ali S., Shah S.S.A., Javed M.S., Najam T., Parkash A., Khan S., Bajaber M.A., Eldin S.M.M., Tayeb R.A., Rahman M.M. (2024). Recent advances of transition metal dichalcogenides-based materials for energy storage devices, in view of monovalent to divalent ions. Chem. Rec..

[87] Ahmed I., Jhung S.H. (2024). Catalytic oxidation reactions for environmental remediation with transition metal nitride nanoparticles. J. Environ. Chem. Eng..

[88] Guo X., Apostol P., Zhou X., Wang J., Lin X., Rambabu D., Du M., Er S., Vlad A. (2024). Towards the 4 V-class n-type organic lithium-ion positive electrode materials: The case of conjugated triflimides and cyanamides. Energy Environ. Sci..

[89] Liang R., Dong J., Li J., Jin H., Wei M., Bai T., Ren W., Xu Y., He B., Suo Z. (2024). DNAzyme-driven bipedal DNA walker and catalytic hairpin assembly multistage signal amplified electrochemical biosensor based on porous AuNPs@Zr-MOF for detection of Pb^2+^. Food Chem..

[90] Ma D., Zhao Z., Wang Y., Yang X., Yang M., Chen Y., Zhu J., Mi H., Zhang P. (2024). Unlocking the design paradigm of in-plane heterojunction with built-in bifunctional anion vacancy for unexpectedly fast sodium storage. Adv. Mater..

[91] Ning J., Zhang X., Xie D., He Q., Hu J., Tang J., Li R., Meng H., Yao K.X. (2024). Unveiling phenoxazine’s unique reversible two-electron transfer process and stable redox intermediates for high-performance aqueous zinc-ion batteries. Angew. Chem. Int. Ed..

[92] Cho J. (2024). Emerging Electrocatalytic Textile Electrodes for Highly Efficient Alkaline Water Electrolysis. ACS Mater. Lett..

[93] He B., Bai F., Jain P., Li T. (2025). A Review of Surface Reconstruction and Transformation of 3d Transition-Metal (Oxy) Hydroxides and Spinel-Type Oxides during the Oxygen Evolution Reaction. Small.

[94] Xu S., Zhang P., Li L., Moon M., Chung C., Li H. (2025). Challenges and Emerging Trends in Hydrogen Energy Industrialization: From Hydrogen Evolution Reaction to Storage, Transportation, and Utilization. Small.

[95] Mafokoane M.A., Ou X., Musyoka N.M., Chang F. (2025). Carbon Dioxide Activation and Hydrogenation into Value-Added C1 Chemicals over Metal Hydride Catalysts. Catalysts.

[96] Weng C., Song Y., Zou K., Zhang Y., Han Z., Ma Y., Chen H., Yang X., Lin W. (2025). Advanced Catalyst Restructuring Strategies for Targeted C1 Production in CO_2_ Electroreduction. Adv. Energy Mater..

[97] Ahmed S., Khan M.K., Kim J. (2025). Revolutionary advancements in carbon dioxide valorization via metal–organic framework-based strategies. Carbon Capture Sci. Technol..

[98] Ahmed S., Hussain M.S., Khan M.K., Kim J. (2025). Innovations in catalysis towards efficient electrochemical reduction of CO_2_ to C1 chemicals. J. Energy Chem..

[99] Yuan Y., Han C., Fu Y., Ye Z., Shen Q., Feng W., Zhao Y. (2024). Structural energy storage composites based on etching engineering Fe-doped Co MOF electrode toward high energy density supercapacitors. J. Power Sources.

[100] Ullah N., Shah S.S., Suliman M.H., Ismail F., Kaci S., Aishah N., Amin S., Usman M. (2025). Electrochemical CO_2_ Reduction: A Review toward Sustainable Energy Conversion and Storage. Energy Fuels.

[101] Zheng Y., Zhang Y., Man Z., Chen W., Lu W., Wu G. (2024). Electrochemical exfoliation and growth of nickel–cobalt layered double hydroxides@black phosphorus heteronanostructure textiles for robust foldable supercapacitors. Adv. Funct. Mater..

[102] Zhao Q., Stalin S., Zhao C.-Z., Archer L.A. (2020). Designing Solid-State Electrolytes for Safe, Energy-Dense Batteries. Nat. Rev. Mater..

[103] Zhang H., Zhang M., Liu R., He T., Xiang L., Wu X., Piao Z., Jia Y., Zhang C., Li H. (2024). Fe_3_O_4_-doped mesoporous carbon cathode with a plumber’s nightmare structure for high-performance Li-S batteries. Nat. Commun..

[104] Yuan K., Lin Y., Li X., Ding Y., Yu P., Peng J., Wang J., Liu H., Dou S. (2024). High-safety anode materials for advanced lithium-ion batteries. Energy Environ. Mater..

[105] Yang Y., Wu C., He X.-X., Zhao J., Yang Z., Li L., Wu X., Li L., Chou S.-L. (2024). Boosting the development of hard carbon for sodium-ion batteries: Strategies to optimize the initial Coulombic efficiency. Adv. Funct. Mater..

[106] Xu Q., Liu Z., Jin Y., Yang X., Sun T., Zheng T., Ning Y.L., Wang T., Li K.W., Jiang J. (2024). A bipolar-type covalent organic framework on carbon nanotubes with enhanced density of redox-active sites for high-performance lithium-ion batteries. Energy Environ. Sci..

[107] Xu M., Zhu X., Lai Y., Xia A., Huang Y., Zhu X., Liao Q. (2024). Production of hierarchical porous bio-carbon based on deep eutectic solvent fractionated lignin nanoparticles for high-performance supercapacitor. Appl. Energy.

[108] Wang L., Fan Z., Yue F., Zhang S., Qin S., Luo C., Pang L., Zhao J., Du J., Jin B. (2024). Flower-like 3D MoS_2_ microsphere/2D C_3_N_4_ nanosheet composite for highly sensitive electrochemical sensing of nitrite. Food Chem..

[109] Wang C., Xie Y., Huang Y., Zhou S., Xie H., Jin H., Ji H. (2024). Li_3_PO_4_-enriched SEI on graphite anode boosts Li^+^ de-solvation enabling fast-charging and low-temperature lithium-ion batteries. Angew. Chem. Int. Ed..

[110] Song X., Li X., Shan H., Wang J., Li W., Xu K., Zhang K., Sari H.M.K., Lei L., Xiao W. (2024). V-O-C bonding of heterointerface boosting kinetics of free-standing Na5V12O32 cathode for ultralong lifespan sodium-ion batteries. Adv. Funct. Mater..

[111] Peng C., Wang F., Chen Q., Yan X., Wu C., Zhang J., Tang W., Chen L., Wang Y., Mao J. (2024). Proton-coupled chemistry enabled p–n conjugated bipolar organic electrode for high-performance aqueous symmetric battery. Adv. Funct. Mater..

[112] Zhu C., Yang G., Li H., Du D., Lin Y. (2015). Electrochemical Sensors and Biosensors Based on Nanomaterials and Nanostructures. Anal. Chem..

[113] Pati J., Dhaka R.S. (2024). Mixed polyanionic NaFe_1.6_V_0.4_(PO4)(SO_4_)_2_@CNT cathode for sodium-ion batteries: Electrochemical diffusion kinetics and distribution of relaxation time analysis at different temperatures. J. Power Sources.

[114] Niu M., Dong L., Yue J., Li Y., Dong Y., Cheng S., Lv S., Zhu Y.-H., Lei Z., Liang J.-Y. (2024). A fast-charge graphite anode with a Li-ion-conductive, electron/solvent-repelling interface. Angew. Chem. Int. Ed..

[115] Muchuweni E., Mombeshora E.T., Muiva C.M., Sathiaraj T.S. (2024). Towards high-performance lithium-ion batteries by introducing graphene-based materials into LiFePO_4_ cathodes: A review. Nano Trends.

[116] Mohammadnezhad K., Ahour F., Keshipour S. (2024). Electrochemical determination of ascorbic acid using palladium supported on N-doped graphene quantum dot modified electrode. Sci. Rep..

[117] Liu J., Di Z., Wan Y., Wang K., Sun W., Dai J., Zhang W., Ran F. (2024). Sub-micron porous Si-C/graphite anode with interpenetrated 3D conductive networks towards high-performance lithium-ion batteries. J. Alloys Compd..

[118] Wu R., Wang Z., Fu Y., Jiang J., Chen Y., Liu T. (2025). High-Sensitive Hydrogel Optofluidic Microcavities for Heavy Metal Ion Detection. ACS Sens..

[119] Mubeen I., Shah S., Pervaiz E., Miran W. (2024). The promising frontier for next-generation energy storage and clean energy production: A review on synthesis and applications of MXenes. Mater. Sci. Energy Technol..

[120] Khan M., Hussain A., Saleh M.T., Ibrahim M., Attique F., Sun X., Unalan H.E., Shafi M., Khan Y., Khan I. (2024). Cutting-edge advancements in MXene-derived materials: Revolutionary electrocatalysts for hydrogen evolution and high-performance energy storage. Coord. Chem. Rev..

[121] He L., Zhuang H., Fan Q., Yu P., Wang S., Pang Y., Chen K., Liang K. (2024). Advances and challenges in MXene-based electrocatalysts: Unlocking the potential for sustainable energy conversion. Mater. Horiz..

[122] Kitchamsetti N., Cho J.S. (2024). A roadmap of recent advances in MXene@MOF hybrids and their derived composites: Synthesis, properties, and utilization as electrodes for supercapacitors, rechargeable batteries, and electrocatalysis. J. Energy Storage.

[123] Mathew S., Sunajadevi K.R.P., Pinheiro D. (2025). Transition metal oxide/chalcogenide-integrated MXene heterostructures: Emerging materials for supercapacitors and water splitting. Mater. Adv..

[124] Vallem S., Venkateswarlu S., Li Y., Song S., Li M., Bae J. (2024). MXene- and MOF-based single-atom catalysts for next-generation battery chemistry: A synergy of experimental and theoretical insights. Energy Storage Mater..

[125] Pramanik A., Sengupta S., Saju S.K., Chattopadhyay S., Kundu M., Ajayan P.M. (2024). Ternary metal sulfides as electrode materials for Na/K-ion batteries and electrochemical supercapacitors: Advances, challenges, and prospects. Adv. Energy Mater..

[126] Sultana S., Hossain R., Jiwanti P.K., Wardhana B.Y. (2023). Recent progress in development of cost-effective and highly efficient Pt-group-metal-free ORR and HER electrocatalysts for next-generation energy devices. J. Electrochem. Soc..

[127] Luo Z., Hossain D., Li Y., Goddard W.A. (2023). Two-dimensional materials-based structure design for energy conversion and storage. ECS Meet. Abstr..

[128] Mkhohlakali A., Ramashala N., Mapukata S., Nyembe S., Hlatshwayo L. (2024). Recent progress on metal hydride and high-entropy materials as emerging electrocatalysts for energy storage and conversion. Hydrides—Preparation, Characterization and Applications.

[129] Duan L., Zhang Y., Tang H., Liao J., Zhou G., Zhou X. (2024). Recent advances in high-entropy layered oxide cathode materials for alkali metal-ion batteries. Adv. Mater..

[130] Zhang Z., Dong J., Huang C., Wan K., Feng Z., Li B. (2025). Recent Progress and Perspectives of Single-Atom Catalysts with Framework Architecture for Zinc-Air Battery Cathodes. Chem. Eng. J..

[131] Cui S., Miao W., Peng H., Ma G., Lei Z., Zhu L., Xu Y. (2023). Covalent organic frameworks as electrode materials for alkali metal-ion batteries. Chemistry.

[132] Loura N., Mor S., Nandal D., Nandal V., Dhull V. (2024). Synthesis strategies of smart 3D nanoarchitectures and their applications in energy storage and conversion. Energy Storage.

[133] Prasankumar T., Kumar S.S.A., Badawi N.M., Kasi R., Subramaniam R.T. (2025). Nanofibers for high-capacity and sustainable energy solutions. Advances in Nanofiber Research.

[134] Wang T., Shi Z., Zhong Y., Ma Y., He J., Zhu Z., Cheng X., Lu B., Wu Y. (2024). Biomass-derived materials for advanced rechargeable batteries. Small.

[135] Sharma G.K., Pramanik A., Puthirath A.B., Chattopadhyay S., Terlier T., Pieshkov T., Saju S.K., Vajtai R., Kaur D., Ajayan P.M. (2024). A flexible Na-ion supercapacitor/battery hybrid device. Adv. Sustain. Syst..

[136] Wang Z., Sun C., Lu L., Jiao L. (2023). Recent progress and perspectives of solid-state Na–CO_2_ batteries. Batteries.

[137] Kadam S.A., Kadam K.P., Ma Y.R. (2024). Recent progress in transition metal nitride electrodes for supercapacitor, water splitting, and battery applications. J. Alloys Compd..

[138] Rani B.J., Sivanantham A., Cho I.S. (2023). Nanostructured spinel manganates and their composites for electrochemical energy conversion and storage. Adv. Funct. Mater..

[139] Kim S., Park G., Lee S.J., Seo S., Ryu K., Kim C.H., Choi J.W. (2023). Lithium Metal Batteries: From Fundamental Research to Industrialization. Adv. Mater..

[140] Yu A., Zhang W. (2024). Molten salt electrolytic CO_2_-derived carbon-based nanomaterials for energy storage and electrocatalysis: A review. ACS Appl. Nano Mater..

[141] Ismail K.M.H., Mahalingam S., Raj B., Kim J. (2024). Carbon fiber-reinforced polymers for energy storage applications. J. Energy Storage.

